# High Prevalence of Genogroup I and Genogroup II Picobirnaviruses in Dromedary Camels

**DOI:** 10.3390/v13030430

**Published:** 2021-03-08

**Authors:** Jade L. L. Teng, Ulrich Wernery, Po Chun Wong, Elaine Chan, Hwei Huih Lee, Sunitha Joseph, Ru Bai, Ying Tang, Emily Y. M. Wong, Susanna K. P. Lau, Patrick C. Y. Woo

**Affiliations:** 1 Department of Microbiology, Li Ka Shing Faculty of Medicine, The University of Hong Kong, Hong Kong, China; llteng@hku.hk (J.L.L.T.); flamingowong@gmail.com (P.C.W.); elaineee@gmail.com (E.C.); cerrapotence@gmail.com (H.H.L.); fionabai314@gmail.com (R.B.); ytang.ashley@gmail.com (Y.T.); emilyhk2811@gmail.com (E.Y.M.W.); skplau@hkucc.hku.hk (S.K.P.L.); 2 Central Veterinary Research Laboratory, P.O. Box 597, Dubai, United Arab Emirates; sjoseph@cvrl.ae

**Keywords:** picobirnaviruses, dromedary, genogroup I, genogroup II, diversity

## Abstract

Picobirnaviruses (PBVs) are small non-enveloped bisegmented double-stranded RNA viruses found in humans, mammals, and birds. Increasing molecular epidemiology studies suggest a high sequence diversity of PBVs in numerous hosts and the environment. In this study, using 229 fecal samples from dromedary camels in Dubai, 52.8% were positive for PBVs, of which 77.7% and 41.3% were positive for genogroup I and II, respectively, and 19.0% were positive for both genotypes. Phylogenetic analysis showed high diversity among the sequences of genogroup I and II dromedary PBVs. Marked nucleotide polymorphisms were observed in 75.5% and 46.0% of genogroup I and II RNA-dependent RNA polymerase (RdRp) sequences, respectively, suggesting the co-existence of multiple strains in the same specimen. Both high genetic diversity and prevalence of genogroup I and II PBV in dromedaries were observed. In fact, the prevalence of genogroup II PBV in dromedaries is the highest among all animals to date. The complete/near-complete core genomes of five genogroup I and one genogroup II dromedary PBVs and partial segment 1 and 2 of both genotypes were also sequenced. The dromedary PBV genome organizations were similar to those of other animals. Genetic reassortment and mutation are both important in the ecology and evolution of PBVs.

## 1. Introduction

Picobirnaviruses (PBVs) are small non-enveloped bisegmented double-stranded RNA viruses found in humans and a wide variety of mammals and birds. Since its first discovery in fecal samples of humans and rats in 1988 [1,2], PBVs have been reported in a variety of other terrestrial mammals, birds, and environmental water samples [3,4,5,6,7,8,9,10,11,12,13,14,15,16,17,18,19,20,21,22,23]. In 2012, we reported the first PBV reported in a marine animal with the discovery of an otarine PBV in a California sea lion from an oceanarium in Hong Kong [20]. Recently, we also described the first discovery of PBVs in fecal samples collected from dromedary camels in the Middle East using a metagenomics study [21].

The genome of a PBV consists of two segments, segment 1 and segment 2. Segment 1 contains the capsid gene and another open reading frame (ORF), which encodes a putative protein of unknown function, whereas segment 2 contains the RNA-dependent RNA polymerase (RdRp) gene [20,24]. By sequence and phylogenetic analyses of RdRp genes, PBVs were classified into genogroups I and II [24,25]. Recently, it was reported that a third genogroup of PBV, genogroup III, may exist [23,26]. In the last few years, we and others have described high prevalence and diversity of genogroup I PBV in marine and terrestrial mammals [12,13,27]. However, no report has described a high prevalence of genogroup II PBV in any mammals. Furthermore, there are far more genogroup I than genogroup II PBV genomes and gene sequences in GenBank. Based on the results of our metagenomic study [21], we hypothesized that a high prevalence and diversity of both genogroup I and genogroup II PBVs may be present in dromedaries. To test this hypothesis, we carried out a molecular epidemiology study on both genogroup I and genogroup II PBVs using fecal samples collected from dromedaries in Dubai, the United Arab Emirates, with two pairs of highly sensitive PCR primers. The genomes of some of the PBV strains were also sequenced and analyzed.

## 2. Materials and Methods

### 2.1. Sample Collection

All fecal samples of dromedaries were leftover specimens submitted for coprological studies to Central Veterinary Research Laboratory in Dubai, the United Arab Emirates, from January to July 2013. A total of 229 dromedaries, including 224 adult dromedaries and 5 dromedary calves, were tested in this study.

### 2.2. RNA Extraction

Viral RNA was extracted from each of the 229 fecal samples using QIAamp viral RNA minikit (Qiagen, Hilden, Germany). The RNA was eluted in 60 μL of AVE buffer (Qiagen, Hilden, Germany) and was used as template for RT-PCR.

### 2.3. RT-PCR for PBVs and DNA Sequencing

PBV screening was performed by PCR amplification of a 205-bp and a 207-bp fragment of RdRp gene of genogroup I and genogroup II PBV, respectively, using degenerate primers (genogroup I: 5′-CAAARTTYGACCARCACTT-3′ and 5′-TCRTCDGCRTTGGTACCACC-3′; genogroup II: 5′-WTGGATGTTTCCGATGTC-3′ and 5′-TGYGCATCCATYTTMGTGGTGTCTC-3′) designed by multiple alignments of the nucleotide sequences of available RdRp genes of PBVs as previously described [27]. Reverse transcription was performed using the SuperScript III kit (Invitrogen, USA) and the reaction mixture (10 μL) contained RNA, first-strand buffer (50 mM Tris-HCl pH 8.3, 75 mM KCl, 3 mM MgCl_2_), 5 mM DTT, 50 ng random hexamers, 500 μM of each dNTPs and 100 U Superscript III reverse transcriptase. The mixtures were incubated at 25 °C for 5 min, followed by 50 °C for 60 min and 70 °C for 15 min. The PCR mixture (25 μL) contained cDNA (1 μL), PCR buffer (10 mM Tris-HCl pH 8.3, 50 mM KCl, 2 mM MgCl_2_), 200 μM of each dNTPs and 1.0 U *Taq* polymerase (Applied Biosystems, USA). The mixtures were amplified in 60 cycles of 94 °C for 1 min, 50 °C for 1 min and 72 °C for 1 min and a final extension at 72 °C for 10 min in an automated thermal cycler (Applied Biosystems, USA). Standard precautions were taken to avoid PCR contamination, and no false-positive was observed in negative controls.

All PCR products were gel-purified using the QIAquick gel extraction kit (Qiagen, Germany). Both strands of the PCR products were sequenced twice with Prism 3730xl DNA Analyzer (Applied Biosystems, Foster City, CA, USA) using the two PCR primers. Sequencing results displayed multiple nucleotide peaks suggesting more than one type of PBV was present in the sample. Hence, the purified PCR products were subsequently cloned using the pCR-II-TOPO TA cloning kit (Invitrogen, USA) according to manufacturer’s instructions. Ten clones were selected for each sample. Both strands of each clone were sequenced using primers 5′-TAATACGACTCACTATAGGG-3′ and 5′-CGGCTCGTATGTTGTGTGGA-3′. The sequences of the clones were compared with known sequences of the RdRp of PBVs in the GenBank database.

### 2.4. Genome Sequencing of Genogroups I and II PBVs

Genome sequencing was performed for five fecal specimens positive for PBV (15C, 17C, 78C, 101C, and 103C). Both genogroup I and II PBVs were detected in samples 78C, 101C and 103C, while only genogroup I PBVs were detected in samples 15C and 17C. Six segment 1 (15C, 17C, 78C, 101C, 103C Sequence Type 1 and 103C Sequence Type 2) and five segment 2 (15C, 17C, 78C, 101C, and 103C) of genogroup I PBVs, and two segment 1 (78C and 101C) and three segment 2 (78C, 101C, and 103C) of genogroup II PBVs detected in dromedary fecal samples were amplified and sequenced using published strategies for double-stranded RNA viruses [28]. RNA was extracted from the original fecal specimens using the EZ1 virus mini kit (Qiagen, Hilden, Germany) and was used as the template for RT-PCR and sequencing. Adaptor primer, with 3′ NH_2_ blocking group, was ligated to the 3′ termini of the viral RNA and subjected to reverse transcription using the complementary primer. After RNA hydrolysis, reannealing and end-filling, single-primer amplification of viral genomic segments was performed using complementary primer and genome-specific primers. Additional primers were designed from the results of the first and subsequent rounds of sequencing. The 5′ and 3′ ends of the viral genomes were confirmed by rapid amplification of cDNA ends using the 5′/3′ RACE kit (Roche, Mannheim, Germany). The PCR products were gel purified and sequenced using an ABI Prism 3700 DNA analyzer (Applied Biosystems, USA). Sequences were assembled and manually edited to produce the final sequences of the viral genomes. The nucleotide sequences were submitted to the DNA Data Bank of Japan under the GenBank accession numbers LC337994 – LC338009.

### 2.5. Genome Analysis

The G+C content of segment 1 and segment 2 sequences and the UTR regions were calculated using BioEdit (v.7.0.9.1) [29]. The open reading frames (ORFs) in each segment were predicted by ORFfinder [30]. The identities of predicted proteins were verified by BLASTP [31]. The conserved motif (ExxRxNxxxE) located in the hypothetical protein of segment 1 was determined by Weblogo 3 [32]. The conserved motifs (D-T/S-D, SG-T, and GDD) located in the RdRp protein of segment 2 were determined by comparing determined amino acid sequences to those of other PBVs. Phylogenetic trees were constructed in MEGA X [33] using the maximum likelihood method based on the Jones-Taylor-Thornton model with uniform rates and bootstrap values calculated from 1000 replicates.

Sequenced genomes were defined as complete if the entire gene was identified, i.e., start and stop codons were located, and conserved bases were identified in both the 5′ and 3′ UTRs. Genomes were defined as near-complete if the entire gene was identified, but conserved bases were not present in either or both 5′ and 3′ UTRs. Genomes were defined as partial if the entire gene was not identified, i.e., the start and/or stop codons were not located.

## 3. Results

### 3.1. Detection of PBVs in Dromedary Fecal Samples and Phylogenetic Analysis

**Figure 1:**
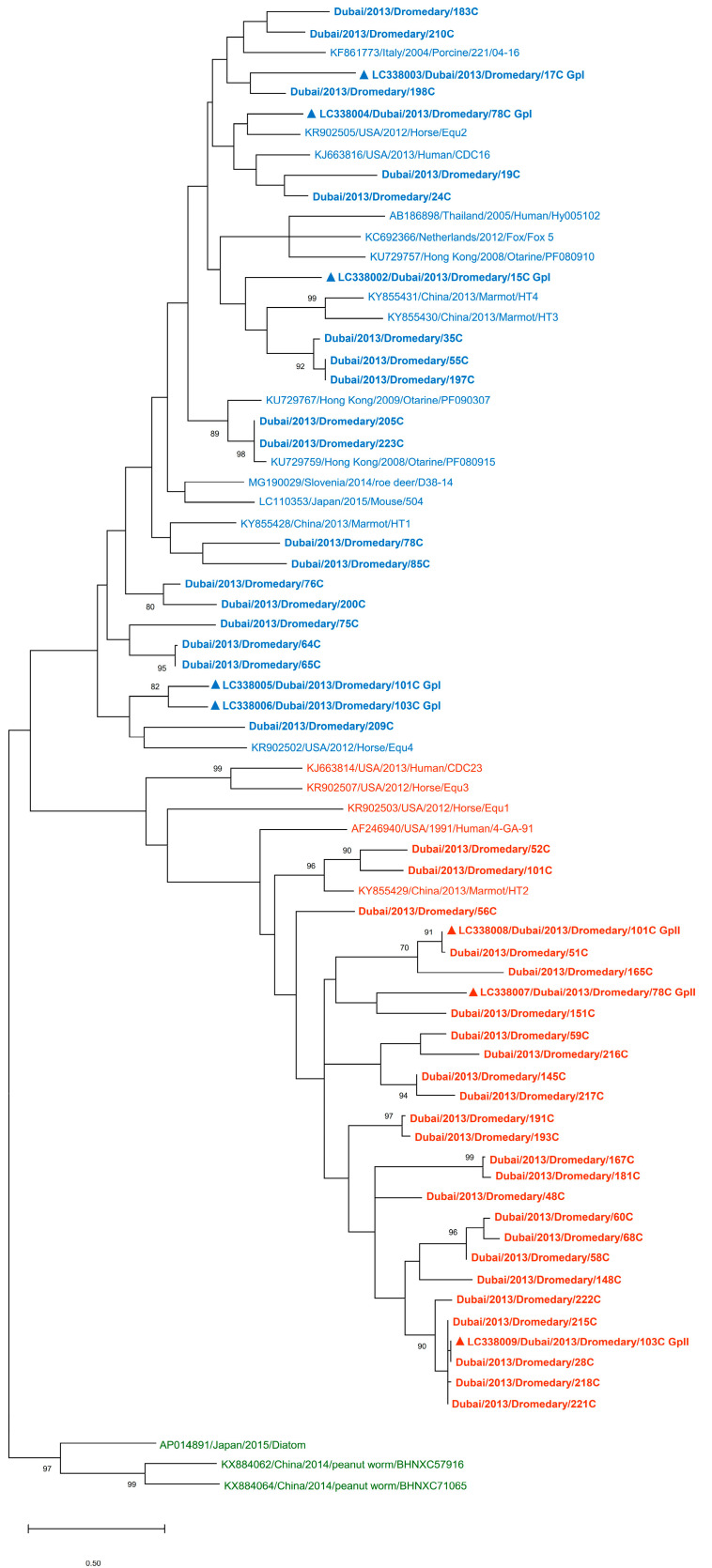
Phylogenetic analysis of the RdRp of genogroup I and genogroup II PBVs and their relationship with other genogroup I, II, and III PBV sequences. Sequences that did not display nucleotide polymorphisms (50 out of 121 PBV-positive samples detected from a screening of 229 dromedary fecal samples) were selected for phylogenetic analysis. The tree was constructed using the maximum likelihood method and is based on the nucleotide of a 205 bp RdRp fragment of segment 2. Numbers at nodes indicated level of bootstrap support calculated from 1000 replicates; bootstrap values below 70% are not shown, and the scale bar indicates the number of nucleotide substitutions per site. Sequences of dromedary samples included in the present study are shown in bold. Sequences of dromedary samples that were further selected for genome sequencing are indicated by a triangle. Reference sequences were selected and classified into their associated genogroups based on phylogenetic analysis of the RdRp by the International Committee on Taxonomy of Viruses (ICTV) [34]. Blue, genogroup I; red, genogroup II; green, genogroup III.

Fecal samples from a total of 229 dromedaries were collected and determined for the presence of PBVs. Overall, 52.8% (121/229) of the fecal samples were positive for PBVs. Of these 121 positive samples, 77.7% (94/121) and 41.3% (50/121) were positive for genogroup I and genogroup II PBVs, respectively (Table 1). Genogroup I and genogroup II PBVs were both detected in 19.0% (23/121) of the positive samples. Of note, PBV was detected only in the adult dromedary samples and not in the dromedary calf samples. Phylogenetic analysis showed that the sequences of both genogroup I and genogroup II PBVs from dromedaries were highly diverse (Figure 1).

Marked nucleotide polymorphisms were observed in 75.5% (71/94) of the genogroup I RdRp sequences, suggesting the possible existence of multiple strains in the same specimen. Sequencing and phylogenetic analysis of the 23 RdRp sequences without polymorphisms revealed that these sequences encompass 45.4% to 98.0% nucleotide identity (2–53 nucleotide difference) to other genogroup I PBV sequences and was most closely related to otarine PBV/GpI (GenBank KU729759), otarine PBV/GpI (GenBank KU729767), human PBV/GpI (GenBank KJ663816), fox PBV/GpI (GenBank KC692366), and horse PBV/GpI (GenBank KR902505) (Supplementary Tables S1 and S2). Similar to genogroup I RdRp, nucleotide polymorphisms were observed in 46.0% (23/50) of the genogroup II RdRp sequences. Sequencing and phylogenetic analysis of the 27 RdRp sequences without polymorphisms revealed that these sequences encompass 15.4% to 55.7% nucleotide identity (37–78 nucleotide difference) to other genogroup II PBV sequences and was most closely related to marmot PBV/GpII (GenBank KY855429) (Supplementary Tables S3 and S4). 

### 3.2. Sequence Analysis of Genogroup I Dromedary PBVs Segments 1 and 2

Genogroup I segment 1 and segment 2 sequences were amplified and sequenced from five dromedary fecal samples (15C, 17C, 78C, 101C, 103C) positive for genogroup I PBV.

#### 3.2.1. Analysis of Six Genogroup I Segment 1 Sequences (One Complete, One Near-Complete, and Four Partial Sequences)

**Figure 2:**
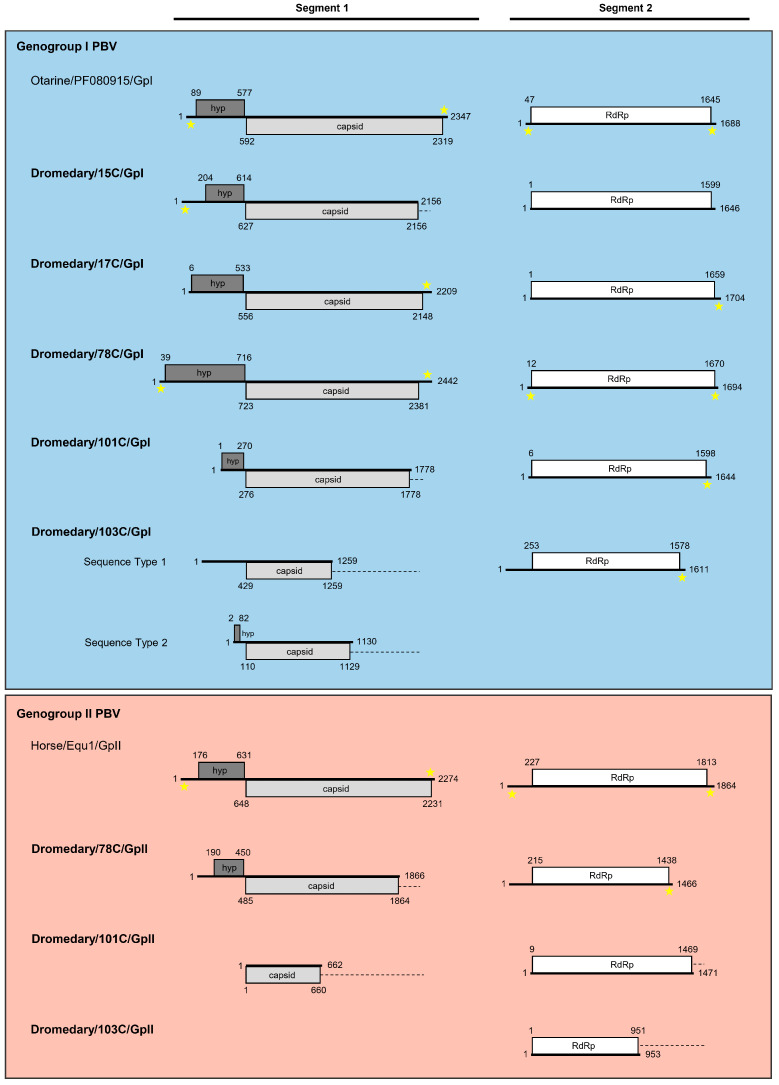
Schematic representation of the gene arrangement of segment 1 and segment 2 of genogroup I (blue box) and II (red box) PBVs sequenced from dromedary fecal samples. Reference sequences are displayed at the top of the corresponding box. The segment 1 sequences in this study encode a capsid protein (light grey box) and an upstream ORF, which encodes a hypothetical protein with unknown function (hyp; dark grey box). The segment 2 sequences encode an RNA-dependent RNA polymerase (RdRp; white box). Stars indicate the presence of conserved bases in the 5′ and/or 3′ UTRs. Partial sequences have dashed lines at the end, indicating the absence of a stop codon. Numbers indicate the position and lengths of the ORFs.

A total of six segment 1 sequences were determined, including one complete segment 1 from 78C, one near-complete segment 1 from 17C, and two partial segment 1 sequences from 15C and 101C, and two partial segment 1 sequences from 103C (Figure 2). The two different partial segment 1 sequences amplified from 103C had 43.0% nucleotide similarity and were designated as Sequence Type 1 and Sequence Type 2. Analysis of the complete and near-complete segment 1 showed that these sequences ranged from 2209 to 2442 bases in length with an overall G+C content of 40.8% to 45.5% (Table 2). All the six amplified segment 1 sequences were found to encompass one long ORF, which encoded for a capsid protein, with complete capsid proteins of 530 and 552 amino acids sequenced from 17C and 78C, respectively. These capsid proteins shared low (13.5% to 41.6%) amino acid identities with those of other genogroup I PBV strains and was most closely related to human PBV/GpI (GenBank number KJ663815), horse PBV/GpI (GenBank number KR902506), and otarine PBV/GpI (GenBank number KU729746) (Supplementary Table S5). Consistent with the organization of segment 1 of other genogroup I PBVs [18,35,36], a short ORF was observed upstream of the capsid protein, with complete sequences of 136 to 225 amino acids determined from 15C, 17C, and 78C (Table 2). This short ORF encodes a hypothetical protein with unknown function and has been shown to harbor a variable number of repeated ExxRxNxxxE motifs [37]. Alignment of the three complete hypothetical protein sequences indicated the presence of 2 to 10 repeated motifs with lengths between the repeats ranging from 1–33 amino acids (Table 3 and Supplementary Figure S1). These findings were comparable to that of segment 1 hypothetical protein sequences observed in other PBVs. Moreover, the 5′ non-coding region (38 bases) of the complete segment 1 (78C) was observed to be AU-rich (G+C content of 26.3%), with five conserved bases GUAAA located at the 5′ end (Table 2). These conserved bases were also found in the partial segment 1 sequence of 15C. The 3′ non-coding region (61 bases) of the complete segment 1 (78C) had a G+C content of 44.3%, with five conserved bases GGAUC located at the 3′ end.

#### 3.2.2. Analysis of Five Genogroup I Segment 2 Sequences (One Complete and Four Near-Complete Sequences)

A total of five segment 2 sequences were determined, including one complete segment 2 from 78C and four near-complete segment 2 from 15C, 17C, 101C, and 103C (Figure 2). Analysis of these segment 2 showed that these sequences ranged from 1611 to 1704 bases in length with an overall G+C content of 41.8 to 45.5% (Table 4). All the five amplified segment 2 sequences were found to encompass one long ORF of 1326 to 1659 bases which encoded RdRps of 441 to 552 amino acids, with complete RdRp sequences determined from 15C, 17C, 78C, 101C, and 103C. These RdRps shared 33.1% to 62.6% amino acid identities with those of other genogroup I PBV strains and was most closely related to otarine PBV/GpI (GenBank number KU729759), horse PBV/GpI (GenBank number KR902505), and otarine PBV/GpI (GenBank number KU729767) (Supplementary Table S6). Three conserved motifs (SG-T, D-T/S-D, and GDD) are commonly located in the RdRp sequences of other dsRNA viruses (Ghosh et al., 2009). These motifs were found in all the five sequenced segment 2 as SGSPYFT, DFSKFD, and GDD, respectively (Supplementary Figure S2). Conserved cysteine and proline residues present in other genogroup I PBV segment 2 sequences [38] were also observed in the five sequenced segment 2 (Supplementary Figure S2). Moreover, the 5′ non-coding region (11 bases) of the complete segment 1 (78C) was observed to be AU-rich (G+C content of 18.2%) with five conserved bases GUAAA located at the 5′ end (Table 4). The 3′ non-coding region (24 bases) of the complete segment 2 (78C) had a G+C content of 45.8% with the bases CCAUU located at the 3′ end. 

### 3.3. Sequence Analysis of Genogroup II Dromedary PBVs Segments 1 and 2

Genogroup II segment 1 and segment 2 sequences were amplified and sequenced from three dromedary fecal samples (78C, 101C, 103C) positive for genogroup II PBVs.

#### 3.3.1. Analysis of Two Partial Genogroup II Segment 1 Sequences 

Two partial segment 1 sequences were determined from 78C and 101C (Figure 2). Analysis of these partial segment 1 showed that these sequences were 1866 and 662 bases in length with a G+C content of 30.4% and 36.9%, respectively (Table 2). The segment 1 sequences were found to encompass one long ORF of 1380 and 660 bases which encoded a partial capsid protein of 459 and 219 amino acids, respectively. The capsid proteins of 78C and 101C shared low (15.2% to 27.1%) amino acid identities with those of other genogroup II PBV strains and was most closely related to horse PBV/GpII (GenBank number KR902504), human PBV/GpII (GenBank number KJ663813) (Supplementary Table S5).

#### 3.3.2. Analysis of Three Genogroup II Segment 2 Sequences (One Near-Complete and Two Partial Sequences) 

A total of three segment 2 sequences were determined, including one near-complete segment 2 from 78C and two partial segment 2 from 101C and 103C (Figure 2). Analysis of the near-complete segment 2 showed that the sequence was 1466 bases in length with an overall G+C content of 40.4% (Table 4). The 3′ non-coding region (28 bases) of the near-complete segment 2 from 78C had a G+C content of 57.1% with the non-conserved bases UUUC located at the 3′ end. All the three segment 2 sequences were found to encompass one long ORF of 951 to 1461 bases which encoded a partial RdRp of 316 to 486 amino acids. These partial RdRp shared 30.5% to 69.9% amino acid identities with those of other genogroup II PBV strains and was most closely related to human PBV/GpII (GenBank number AF246940) and marmot PBV/GpII (GenBank number KY855429) (Supplementary Table S6). The conserved motifs DTTKMD and SGYPRFRR were found in all the three sequenced segment 2 and GDD was located only in 78C and 101C as the partial sequence of 103C did not cover this region (Supplementary Figure S2). However, in contrast to the segment 2 of genogroup I PBVs, only conserved proline residues but not cysteine residues, were observed in all the three sequenced genogroup II PBV segment 2. This coincides with the absence of conserved cysteine residues in other genogroup II PBV segment 2 sequences.

### 3.4. Phylogenetic Analysis of Capsid and RdRp Sequences of Dromedary PBVs

**Figure 3:**
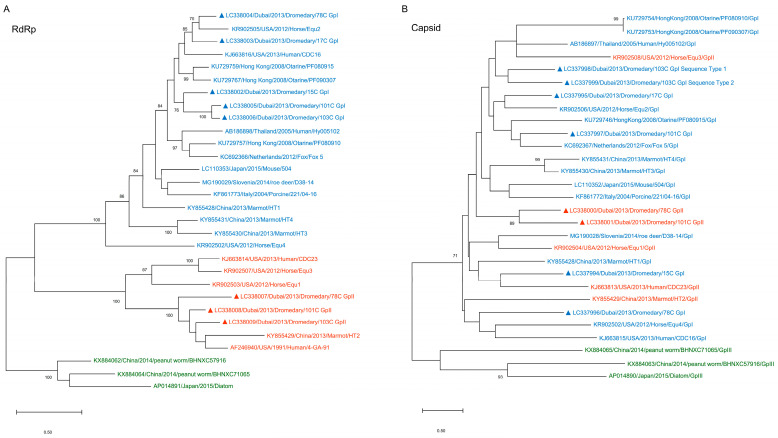
Phylogenetic analysis of (**A**) the RdRp and (**B**) the capsid protein of genogroup I and II PBVs detected in dromedary fecal samples and their relationship with other corresponding protein sequences of genogroup I, II, and III PBVs. The tree was constructed using the maximum likelihood method and is based on the amino acid of a 205 aa RdRp fragment of segment 2 and 166 aa capsid protein fragment of segment 1. Numbers at nodes indicated level of bootstrap support calculated from 1000 replicates; bootstrap values below 70% are not shown, and the scale bar indicates the number of amino acid substitutions per site. Sequences of dromedary samples included in the present study are indicated by a triangle. Blue, genogroup I; red, genogroup II; green, genogroup III.

Phylogenetic analysis of the RdRp and capsid sequences showed different topologies. The RdRp sequences were the basis for genogroup classification and therefore fell into three distinct clusters, genogroups I, II, and III (Figure 3A). However, a corresponding clustering into the three genogroups was not observed in the phylogenetic tree constructed using the capsid sequences (Figure 3B). Instead, the capsid sequences from the different genogroups were all inter-mixed.

## 4. Discussion

In this study, we presented a comprehensive analysis on the molecular epidemiology and genomics of PBVs in dromedaries. In our previous metagenomic study on pooled dromedary fecal samples, we observed an exceptionally higher number of PBV compared to other viral sequences [21]. In the present study, we used two pairs of primers based on conserved regions of the RdRp gene to detect genogroup I and genogroup II PBVs separately in 229 dromedary fecal samples from Dubai. It was observed that genogroup I and genogroup II PBVs were present in 41% and 22% of the samples, respectively, while both genogroup I and II PBVs were present in 10% of the samples. In the literature, the prevalence of genogroup I PBVs in the fecal samples from a wide range of animal species, such as roe deer, pigs, rats, sheep, cattle, chickens, and mongooses, have been studied [9,10,12]. The highest prevalence was in roe deer (62%) and monkeys (48%) [12,19]. However, comprehensive studies on genogroup II PBVs are relatively few compared to genogroup I PBVs and the reported prevalence of genogroup II PBVs in fecal samples of pigs, cattle, and sheep were all <10% [3,4,14,22]. The present study is the first one that described such an unprecedentedly high prevalence of 22% for genogroup II PBVs in animals. We speculate that the relatively higher prevalence of genogroup II PBVs in the present study, as compared to those described in the literature, may be due to the screening primers that we used. This highly sensitive primer set will be used for molecular epidemiology studies of other animals to see if a higher prevalence of genogroup II PBV will also be observed. 

In addition to the high prevalence, a high genetic diversity of genogroup II PBV in dromedaries was also observed. In contrast to genogroup I PBVs of which the genomics and phylogenies have been quite well studied, our knowledge on genogroup II PBVs are relatively scarce due to the small number of genogroup II PBV sequences available. Although there are >2400 PBV nucleotide sequences available in GenBank, there is significantly less literature relating to genogroup II PBV. In this study, after determining the prevalence, we used the RdRp gene sequences to examine the phylogenetic relationships of dromedary PBVs with other PBVs. Similar to genogroup I dromedary PBVs, genogroup II dromedary PBV strains were also highly diverse (Figure 1). This indicated that there are many strains of both genogroup I and II PBVs circulating in dromedaries. This phenomenon of high diversity has also been observed in most of our previous studies on cattle, horses, pigs, and California sea lions [22,27]. This is different, however, from what we found in monkeys in Hong Kong, in which a few clusters of PBVs were observed [22]. We speculate that this is because most monkeys in Hong Kong live as geographically separated troops, with minimal direct contact among the different troops, and the samples collected in that study was from two separated troops, therefore giving rise to the apparently distinct phylogeny in PBVs from monkeys in Hong Kong [22]. Nevertheless, the high diversity and high prevalence of PBV in numerous hosts suggest a high potential for the virus to further spread and cause disease in other animal species or for more virulent forms of the virus to transmit into natural hosts such as humans [39,40].

The genome organizations of both genogroup I and II PBVs in dromedaries are similar to those of other animals. In this study, after the initial molecular epidemiology and phylogenetic analysis, we attempted to sequence the core genomes (segment 1 and segment 2) for a number of dromedary PBV strains using the chromosomal walking approach for subsequent in-depth genome analysis. The complete or almost complete core genomes of five strains of genogroup I dromedary PBV, but only one strain of genogroup II PBV, were sequenced. In addition, four partial segment 1 of genogroup I dromedary PBV and two partial segment 1 and two partial segment 2 of genogroup II dromedary PBV, respectively were successfully obtained. We speculate that the difficulty in sequencing genogroup II PBV genomes is due to the high genetic diversity of PBVs as well as the relatively small number of genogroup II genomes and sequence fragments in GenBank; and therefore, difficult to design degenerate primers for chromosomal walking. This is similar to the difficulty in designing screening primers for the detection of genogroup II PBVs as we mentioned above. In fact, only four complete/near-complete segment 1 and five complete segment 2 sequences of genogroup II PBV sequences were available. The genetic content of both genogroup I and II dromedary PBVs were observed to be consistent with previously described PBV genomes, i.e., a capsid protein and an upstream ORF encoding a putative protein of unknown function on segment 1, and an RdRp gene in segment 2. The putative protein of unknown function shows no homology with any other proteins in GenBank and does not possess any known protein domain or functional site. Further experiments will be needed to examine whether it is expressed and its function in PBV.

Genetic reassortment and mutation are both important in the evolution of PBVs. The phylogenetic trees constructed using RdRp and capsid sequences showed completely different topologies (Figure 3A,B). This indicates that similar to influenza viruses, in which reassortment of the different segments is important in the generation of new strains, reassortment of the two segments in PBVs has also played a significant role in its evolution. This is supported by the observation that the same dromedary may be infected with more than one PBV, as noted by the presence of nucleotide polymorphisms, and even two different genogroups of PBV, which is a prerequisite for reassortment to take place. In addition to reassortment, the high number of nucleotide polymorphism in both genogroup I and II PBV sequences found in the same dromedary showed that PBVs, in general, have high frequencies of mutation. These indicate that both reassortment of the two gene segments, as well as mutation, are both crucial in shaping the PBV genomes and their phylogenies that we now observe.

## Figures and Tables

**Table 1:** Prevalence of genotype I and genotype II picobirnaviruses (PBVs) in 121 dromedaries positive for the presence of PBV as detected by RT-PCR of partial fragments of RNA-dependent RNA polymerase (RdRp) gene, and the number of positive PBV samples with and without the presence of nucleotide polymorphism.

Presence/Absence of Polymorphism	Number (%) of Samples Positive for:	
	Genogroup I PBV	Genogroup II PBV
	94/121 (77.7)	50/121 (41.3)
With polymorphism	71/94 (75.5)	23/50 (46.0)
Without polymorphism	23/94 (24.5)	27/50 (54.0)

**Table 2:** Genomic features of segment 1 sequences of genogroup I and genogroup II PBVs from dromedary fecal samples.

PBV Strain			ORF Features					5′ UTR Features			3′ UTR Features		
	Length (nt)	G+C Content (%)	Protein	Location (nt)	Length (nt)	Length (aa)	Frame	Length (nt)	G+C Content (%)	5′ Bases	Length (nt)	G+C Content (%)	3′ Bases
*Genogroup I PBV*													
^a^ 15C/GpI	2156	44.3	hypothetical	204–614	411	136	3	203	41.4	GUAAA	-	-	-
			capsid	627–2156	1530	509	3						
^b^ 17C/GpI	2209	45.5	hypothetical	6–533	528	175	3	-	-	-	61	45.9	CCUGC
			capsid	556–2148	1593	530	1						
^c^ 78C/GpI	2442	40.8	hypothetical	39–716	678	225	3	38	26.3	GUAAA	61	44.3	GGAUC
			capsid	723–2381	1659	552	3						
^a^ 101C/GpI	1778	43.6	hypothetical	1–270	270	89	1	-	-	-	-	-	-
			capsid	276–1778	1503	500	3						
^a^ 103C/GpI (ST1)	1259	39.8	hypothetical	-	-	-	-	-	-	-	-	-	-
			capsid	429–1259	831	276	3						
^a^ 103C/GpI (ST2)	1130	38.9	hypothetical	2–82	81	26	2	-	-	-	-	-	-
			capsid	110–1129	1020	339	2						
*Genogroup II PBV*													
^a^ 78C/GpII	1866	30.4	hypothetical	190–450	261	86	1	-	-	-	-	-	-
			capsid	485–1864	1380	459	2						
^a^ 101C/GpII	662	36.9	hypothetical	-	-	-	-	-	-	-	-	-	-
			capsid	1–660	660	219	1						

^a^ partial sequence; ^b^ near-complete sequence; ^c^ complete sequence; GpI: genogroup I; GpII: genogroup II; ST: sequence type; -: data not available.

**Table 3:** Number of repeated ExxRxNxxxE motifs and the lengths between the repeats in segment 1 hypothetical protein sequences of genogroup I and genogroup II PBVs detected from dromedary fecal samples compared to other PBV strains.

PBV Genome	Length (aa)	Number of Repeated Motifs	Length between Repeats (aa)
^a^ LC337994/Dromedary/15C/GpI	136	7	1–8
^a^ LC337995/Dromedary/17C/GpI	175	2	1
^a^ LC337996/Dromedary/78c/GpI	225	10	1–33
^a,b^ LC337997/Dromedary/101C/GpI	89	1	0
^a,b^ LC338000/Dromedary/78C/GpII	86	2	8
KU729746/Otarine/PF080915/GpI	194	5	1–44
KU729754/Otarine/PF080910/GpI	223	9	1–22
NC007026/Human/Hy005102/GpI	224	5	1–19
KY855431/Marmot/HT4/GpI	190	2	8
KY855430/Marmot/HT3/GpI	184	2	8
LC110352/Mouse/504/GpI	241	7	1–44
KR902502/Horse/Equ4/GpI	212	3	8
KC692367/Fox/Fox_5/GpI	201	7	1–15
KF861772/Porcine/221/04–16/GpI	199	4	8–22
KR902506/Horse/Equ2/GpI	222	6	1–45
KJ663813/Human/CDC23/GpII	116	5	1–12
KR902504/Horse/Equ1/GpII	151	3	8–15
KR902508/Horse/Equ3/GpII	251	4	1–26
KY855429/Marmot/HT2/GpII	311	2	8

^a^ genomes sequenced in this study; ^b^ partial sequence; GpI: genogroup I; GpII: genogroup I.

**Table 4:** Genomic features of segment 2 sequences of genogroup I and genogroup II PBVs from dromedary fecal samples.

PBV Strain			ORFs Features					5′ UTR Features			3′ UTR Features		
	Length (nt)	G+C Content (%)	Protein	Location (nt)	Length (nt)	Length (aa)	Frame	Length (nt)	G+C Content (%)	5′ Bases	Length (nt)	G+C Content (%)	3′ Bases
*Genogroup I PBV*													
^a^ 15C/GpI	1646	44.4	RdRp	1–1599	1599	532	1	-	-	-	-	-	-
^a^ 17C/GpI	1704	45.5	RdRp	1–1659	1659	552	1	-	-	-	45.0	46.7	CUGC
^b^ 78C/GpI	1694	41.8	RdRp	12–1670	1659	552	3	11.0	18.2	GUAAA	24.0	45.8	CCAUU
^a^ 101C/GpI	1644	44.3	RdRp	6–1598	1593	530	3	-	-	-	46.0	47.8	CUGC
^a^ 103C/GpI	1611	43.4	RdRp	253–1578	1326	441	1	-	-	-	33.0	48.5	CUCA
*Genogroup II PBV*													
^a^ 78C/GpII	1466	40.4	RdRp	215–1438	1224	407	2	-	-	-	28.0	57.1	UUUC
^c^ 101C/GpII	1471	42.0	RdRp	9–1469	1461	486	3	-	-	-	-	-	-
^c^ 103C/GpII	953	45.2	RdRp	1–951	951	316	1	-	-	-	-	-	-

^a^ near-complete sequence; ^b^ complete sequence; ^c^ partial sequence; GpI: genogroup I; GpII: genogroup II; -: data not available.

## Data Availability

The reference for data access is indicated in Section 2.4.

## Supplementary Materials

The following are available online at https://www.mdpi.com/1999-4915/13/3/430/s1, Figure S1. Multiple alignment of amino acid sequences of the hypothetical protein from segment 1 of genogroup I and genogroup II PBVs detected in dromedary fecal samples and other representative PBV sequences with the repeated ExxRxNxxxE motif highlighted in grey. Figure S2. Multiple alignment of amino acid sequences of RdRp from segment 2 of genogroup I and genogroup II PBVs detected in dromedary fecal samples and other representative PBV sequences with the conserved motifs D-T/S-D, SG-T, and GDD shown in red boxes (genogroup I), blue boxes (genogroup II) and green boxes (genogroup III), and conserved proline and cysteine residues highlighted in yellow. Table S1. Comparison of pairwise nucleotide identity between genogroup I PBVs identified from dromedaries with known PBV sequences. Table S2. Comparison of pairwise nucleotide difference between genogroup I PBVs identified from dromedaries with known PBV sequences. Table S3. Comparison of pairwise nucleotide identity between genogroup II PBVs identified from dromedaries with known PBV sequences. Table S4. Comparison of pairwise nucleotide difference between genogroup II PBVs identified from dromedaries with known PBV sequences. Table S5. Comparison of pairwise amino acid identity of capsid protein between genogroup I and II PBVs identified from dromedaries with known PBV sequences using 166 aa of the protein. Table S6. Comparison of pairwise amino acid identity of RdRp between genogroup I and II PBVs identified from dromedaries with known PBV.
