# Supplementary material for: High Prevalence of Genogroup I and Genogroup II Picobirnaviruses in Dromedary Camels
Source: Viruses. 2021 Mar 8;13(3):430. doi: 10.3390/v13030430 (PMC7999184; doi:10.3390/v13030430)
Supplement: Supplementary file 1 [file viruses-13-00430-s001.zip › Supp Fig S1.pdf]

Supplementary Figure S1. Multiple alignment of amino acid sequences of the hypothetical protein from segment 1 of genogroup I and genogroup II PBVs detected in dromedary fecal samples and other representative PBV sequences with the repeated ExxRxNxxxE motif highlighted in grey. GpI, genogroup I; GpII, genogroup II; GpIII, genogroup III.

```
                                ....|....| ....|....| ....|....| ....|....| ....|....| ....|....| ....|....| ....|....| ....|....| ....|....| ....|....| ....|....| ....|....|
                                        10         20         30         40         50         60         70         80         90        100        110        120        130
LC337994/Dromedary/15C/GpI      ---------- ---------- ---------- ---------- ------MTQM QLQYHTLQET KRSNRAREDD EDVK------ ---------- ---NRETQRH NVATEGISMN TLSETVRSNR AKEF------
LC337995/Dromedary/17C/GpI      ---------- ---------- ---------- ---------- --------MI EVKQLTLLER RYSTMTANQI ALAKVRE--- --D------A WYHEADIRTR NVANQISAER NMVSLLEAQV AQQN-----A
LC337996/Dromedary/78c/GpI      --MTDLQIKY QQHLETKRSN LANEAETRRS NLAREGKTHR SNVARESEER RYHTLVMTET VRSNMARENE NRIHNRA--- --QEML---G FANLQETSRH NAAQESLGFA QLSETRRKND LDYIVKMGSN
LC337997/Dromedary/101C/GpI     ---------- ---------- ---------- ---------- ----MNASTN AVNSATNARN ATTNFISVSQ ---------- ---------- ---------- ---------- ---------- ----------
LC338000/Dromedary/78C/GpII     ---------- ---------- ---------- ---------- ------MTGN QIAYWQLQET KRSNLINEGL KSEN------ ---------- ---LQETKRS NMRNEDI--- ---------- ----------
KU729746/Otarine/PF080915/GpI   MYFHHGQIYN GCVHVSTVCT ITFILLERR- ---------- ------ISSM TTNQINFFRT QNEKLAAERS AQQHERE--- --LNEIRRSN LAKEQEMRRS NMAKENLGFQ QFNESLRHNK AT-------E
KU729754/Otarine/PF080910/GpI   ---------- ---------- ---------- ---------- ------MTKN QIEYLKLRET QRANLVQEDL TRSRDSR--- --SYEI---G LGTLAESQRH NRATEEQARV SLDETVRHNL AG-------E
AB186897/Human/Hy005102/GpI     ---------- ---------- ---------- ---------- ------MTAN QIAYQKHLET ARVNAVGEMQ RGLE------ ---------- ---LDESRRH NISQEQLKTR ELTELERSNR AVE------K
KY855431/Marmot/HT4/GpI         --MKGGQH-- ---------- ---------- ---------- ------MTTM ELRAVELEEM KRHNLAMERL QDVQ------ ---------- ---MGETARH NLRQESIAHQ ANVNDFTKNS QTYS-----L
KY855430/Marmot/HT3/GpI         ---------- ---------- ---------- ---------- ------MTTL ELQANELAET IRHNKELEKL QGSQ------ ---------- ---LGELNRH NVVQEGIGYQ SNVNDMTRNS QNYA-----V
LC110352/Mouse/504/GpI          --MTRNQIAY WELHETNRAN RARE------ ---------- ------VETN RNNLAVETET NRANLERERM NRVQSNR--- -----NYEVA LRNLFEQRRA TKVRESQSQQ QIEETIRHNR QT-------E
KR902502/Horse/Equ4/GpI         ---------- ---------- ---------- ---------- ------MHEI QIKRADYEES KRHNLAVEDY NNQS------ ---------- ---MQETKRH NVATEQYSTQ SLAETTRHNK AS-------E
KC692367/Fox/Fox_5/GpI          --MTANLISY WRHKEDSRHN ---------- ---------- ------RETE SLNRDVHAEN KRHNLETESH NRNVLEY--- ---------N YVALEETKLH NRTTEAIGWE TAYEQIRHNQ AQEALTSQQI
KF861772/Porcine/221/04-16/GpI  --MLSTQVQY WANKEQARHN ---------- ---------- ------LAME RLTHRQTNES IRHNLATESA TQRQLNL--- --QLQANTNN YRAVTETIRH NRQSESLSWF NAVESRRHNI RQEN-----I
KR902506/Horse/Equ2/GpI         --MTHNQIEY WKN------- ---------- ---------- ------EESK KHNRATESEN YRHDVVSERE TQRHNQQ--- --TEVQ---A LSELNETSRH NKAGET---- ---ETVRHNK AG-------E
KJ663813/Human/CDC23/GpII       ---------- ---------- ---------- ---------- ---------M TCQLYQLLVW RKSGMTRNQI ---------- ---------A YRELLETERS NKARET---- ---------- ----------
KR902504/Horse/Equ1/GpII        ---------- ---------- ---------- ---------- ------MTTN QVNFWKLQEE IKHNRNTESL TKSDLDI--- ---------K QGNLDESIRH NQVNEALNQV SYEEGVRHN- ---------K
KR902508/Horse/Equ3/GpII        ---------- ---------- ---------- ---------- ------MTQN QIAFRNAKEN ERHNLATEA- ---------- ---------- -----ENTRH NSQTEAAQWQ SNVLADQHYQ RQ-------D
KY855429/Marmot/HT2/GpII        ---------- ---------- ---------- ---------- -----MKSSV CFKQFILFTD YFTALRIEDI RRLSANSFKG GCVMTVPQIR YREYREQARH NEATELLGTR QLAETQRTNL ANEAIREEAN

                                ....|....| ....|....| ....|....| ....|....| ....|....| ....|....| ....|....| ....|....| ....|....| ....|....| ....|....| ....|....| ....|....|
                                       140        150        160        170        180        190        200        210        220        230        240        250        260
LC337994/Dromedary/15C/GpI      ---------- -ETNRSNVAK ETETNRANLA KETETYRS-- ---------- ---------- ---------- --------NV AKERET---- ---------- -VRSNMANE- ---------- ----------
LC337995/Dromedary/17C/GpI      RTREREVA-- -ATERRNEIS AQEMQYNYIL QSSRIAEEQR H--------- ---------- ---------- --------NL AAEAET---- ---------- -QRSNTVGEW Q--------- --RTAQRQ--
LC337996/Dromedary/78c/GpI      KIEAAKML-- -ETARANKAR EIETIRSNRA KEMETTRS-- ---------- ---------- ---------- --------NI ARETEA---- ---------- -IRSNIARE- ---------- --QEQYRS--
LC337997/Dromedary/101C/GpI     ---------- -LHERENRKR RNQDFR---- ---------- ---------- ---------- ---------- --------EA ERNAET---- ---------- -RRHNMATE- ---------- ----------
LC338000/Dromedary/78C/GpII     ---------- ---------- ---------- ---------- ---------- ---------- ---------- --------KE DTHDET---- ---------- ---------- ---------- ----------
KU729746/Otarine/PF080915/GpI   SQASSQLR-- -EIERHNRAS VGLGYSNLSE QHRHNVASNS L--------- ---------- ---------- --------GY ANQFEQ---- ---------- -NRHDVAQE- ---------- ----------
KU729754/Otarine/PF080910/GpI   TLTRGNLD-- -ESIRHNQAV EAEASRHNKV GESY-NERAL KESQRHNVAQ EGISRSQVGA SYANINLGYS QLGETTRTNI ARETEN---- ---------- -MRSNVARE- ---------- --QENLRH--
AB186897/Human/Hy005102/GpI     ETSRHNVVTE TETRRSNLAR EWETYRSNSA REME-TQRSN ISYEAIKRGQ LALDRA---- ---------- --------EL NESIRA---- ---------- -TNENLALQY SKLQTESLLT QRGQDLQHKN
KY855431/Marmot/HT4/GpI         GMRNAGIS-- -AMNAQSTAI QAQAFADNVT SQVA-DRVVN QGISQQNAD- ---------- ---------- --------TN ATNAET---- ---------- -NRSNWLTTN DLA------- --GANIAQ--
KY855430/Marmot/HT3/GpI         GMRNAAVN-- -ERNATSNAI QADAMKRNVE SQIT-DRTVN QGINQQNAD- ---------- ---------- --------TN EYNADT---- ---------- -NRSNWLSYN EVN------- --ASNIAL--
LC110352/Mouse/504/GpI          SVAKQQSDIS VRSVTVQEKA QAETRRANMQ QEALNQRKQD EVERLN---- ---------- ---------- --------KT AQRLDA---- ---------- -YRLNEVQRN NKFNNLMQFK TISETERS--
KR902502/Horse/Equ4/GpI         KLTQYVADKN YSATKYSADK NYQGTKYSAD KNYRATKYAA DMNYAGTRY- ---------- ---------- --------AA DSNYAA---- ---------- -AKYSANANL SATQYLANSN LQGTMYTA--
KC692367/Fox/Fox_5/GpI          MLGQQQLA-- -EQRRANQAR EAEIYRSNRV HEIETRRS-- ---------- ---------- ---------- --------NV ARENES---- ---------- -RRHNESLET ---------- --LESRRVFT
KF861772/Porcine/221/04-16/GpI  AATQSYSA-- -LLNAQSQMK NAETQRYRAA TDRY-AAITT AN-------- ---------- ---------- --------LG SYNAFT---- ---------- -NRMNAYTN- ---------- -----QRQ--
KR902506/Horse/Equ2/GpI         AATRYANK-- -ETKRHNKAT EKETKRSNKA SESN-YRKQI EANYATGMAN AGAGYASA-- ---------- --------NA AYQNAI---- ---------- -YTNQHYQRT D--------- --AESKRH--
KJ663813/Human/CDC23/GpII       ---------- -ETSRSNLAN ESENRRSNIA KETETNRS-- ---------- ---------- ---------- --------NT ARESET---- ---------- -NRTNVANE- ---------- ----------
KR902504/Horse/Equ1/GpII        QMEQANMI-- -QANASASQA ASAADRAQAD VSLT-NQK-- ---------- ---------- ---------- --------LL TEEQNT---- ---------- -AKVAAQK-- ---------- ----------
KR902508/Horse/Equ3/GpII        KIAVYNAR-- -EAQRHNAAM EAETNRSNTE READTDAARN QGWYSIGLGA AGAAVGGAAT ---------- --------AI AKGLMN---- ---------- -NKHKNNKNG GSNGGNSSGG EYQGTDPHAR
KY855429/Marmot/HT2/GpII        RINRYGIDVG AETARATAEI AAEASRYSAD RHYDASVYSA NAHTLASQYA ADSSRAAAKY SADQALTAAE YQAQLNSENV KYSADSSAAA SRYSANANVF ASHERTSSEY AQRQAELEFQ REKTQFEN--

                                ....|....| ....|....| ....|....| ....|....| ....|....| ....|....| ....|....| ....|....| ....|....| ....|....| ....|....| ....|....| ..
                                       270        280        290        300        310        320        330        340        350        360        370        380
LC337994/Dromedary/15C/GpI      ---------- ---------- ---------- --ALTRSANR NKAAAD---- YLKVAVSAIP GTS------- ---------- ------VI-- ---------- --GRILG--- ---------- --
LC337995/Dromedary/17C/GpI      ---------- ------DEYN LGVLNI---- -----QQKQR ETQSQE---- KRSAWQLGAS LLN------- -----TVG-- -----GAL-- ---------- --TKLVALW- ---------- --
LC337996/Dromedary/78c/GpI      ---------- ------NLAK E--------- -----KETER HNKKGE---- FIDLVNTGVK AYS------- -----AWSKT GTDTLHAI-- ---------- --GEIIPF-- ---------- --
LC337997/Dromedary/101C/GpI     ---------- ---------- ---------- -----DETRR KNTADM---- WLGSINAAAN TM-------- ---------- -----KGV-- ---------- --GSIIPG-- ---------- --
LC338000/Dromedary/78C/GpII     ---------- ---------- ---------- -----KLNNK HNRVIG---- YINASANVIK SA-------- ---------- ---------- ---------- --GSLANPLN MLFKS----- --
KU729746/Otarine/PF080915/GpI   ---------- ---------- ---------- -----LESQR HNTTTE---- QTSNFGTIGN VLL------- ---------- ------GV-- ---------- --GGLFTKSN KKLKR----- --
KU729754/Otarine/PF080910/GpI   ---------- ------NEST EAINMYRNRA DSSYQKNSLK QGDKRL---- RLDTWKTVFE GAD------- -----TVF-- -----KGI-- ---------- --RNVVPLWL K--------- --
AB186897/Human/Hy005102/GpI     AIIGASA--- ------NAFG SLLGYSTASA DRASREEIAS ANRKSQEHIA SMQVLGSMAN TMF------- -----SSVS- -----NLV-- ---------- --GKTAGAFA GGLS------ --
KY855431/Marmot/HT4/GpI         ---------- ------AEAQ TDLLRA---- ----QEDRAK TDNTLA---- WLKAPGQIFS EWM------- -----GGV-- -----NQL-- ---------- --SNAVPIWG LLTK------ --
KY855430/Marmot/HT3/GpI         ---------- ------NYAN QQLAAA---- ----QADKAN ADKTLS---- WIKAPGQIFN DYM------- -----SGV-- -----NQL-- ---------- --SNAVPIWG LLTK------ --
LC110352/Mouse/504/GpI          ---------- ------NRSN ESIKRINNNI NMRKLSEDVR HNKALE---- FESARHNLSQ EAQ------- -----GYAKL VVDTARSI-- ---------- --FGTLGSLK LGGAR----- --
KR902502/Horse/Equ4/GpI         ---------- ------NQRK ESAKYAADMS SKTSIQNTKT RNATEK---- YKANINGVGN MV-------- -----AAAGA AIGKNVAK-- ---------- --NGKIPIHG TKKGKDL--- --
KC692367/Fox/Fox_5/GpI          ---------- ------SQRS QDLAES---- ---RFELEKT KYQTDV---- FFDAMDMGFD AA-------- ---------- -----KIV-- ---------- --TGLFNTGN KKKGA----- --
KF861772/Porcine/221/04-16/GpI  ---------- ------VTST ESLNSA---- ----RITQMQ HETFQG---- YMNSITNMVT RTS------- -----DSASS WM---RAL-- ---------- --RPWIP--- ---------- --
KR902506/Horse/Equ2/GpI         ---------- ------NEAT ETYSNAKNKI D--LIKNQTQ RDQFSE---- QLDLYWAQYN EAV------- -----RSNNM KLAE-NIM-- ---------- --SNLTKIGV EAAKMGTEKK --
KJ663813/Human/CDC23/GpII       ---------- ------SLRD AEL------- -----QQKKK YEKQRN---- LLEAVKIGTN FI-------- ---------- ---------- ---------- --GNFIP--- ---------- --
KR902504/Horse/Equ1/GpII        ---------- ---------- ---------- -----LSAQN EASISQ---- KQNAWWEISN ALS------- -----LGSGV VGTAAKLV-- ---------- --SHK----- ---------- --
KR902508/Horse/Equ3/GpII        TKSSPTDYGV PLAEYPSLPN DGWAYNPAGA GTAESSVQSG INNARA---- LEQGWITTAL GAL------- -----AAYGV REAEIKREVS DALSKIPAPE PNAGWVTIGS LPMMPGIGVP LF
KY855429/Marmot/HT2/GpII        ---------- ------NLKT QTLRLDQLNS LYNRRHTDAD THRIQAQTSQ TIAATENMVK EFALKARAQN NADELNKANV LLTQMRTLES GIKTVDTFSQ TVERCINLMK EVLP------ --
```
