# Supplementary material for: High Prevalence of Genogroup I and Genogroup II Picobirnaviruses in Dromedary Camels
Source: Viruses. 2021 Mar 8;13(3):430. doi: 10.3390/v13030430 (PMC7999184; doi:10.3390/v13030430)
Supplement: Supplementary file 1 [file viruses-13-00430-s001.zip › Supp Fig S2.pdf]

Supplementary Figure S2. Multiple alignment of amino acid sequences of RdRp from segment 2 of genogroup I and genogroup II PBVs detected in dromedary fecal samples and known PBV sequences with the conserved motifs D-T/S-D, SG-T, and GDD shown in blue boxes (genogroup I), red boxes (genogroup II) and green boxes (genogroup III), and conserved proline and cysteine residues highlighted in yellow. GpI, genogroup I; GpII, genogroup II; GpIII, genogroup III.

```
                                ....|....| ....|....| ....|....| ....|....| ....|....| ....|....| ....|....| ....|....| ....|....| ....|....| ....|....| ....|....| ....|....|
                                        10         20         30         40         50         60         70         80         90        100        110        120        130
LC338002/Dromedary/15C/GpI      ---------- -----MPKNN ETK--FGNYF -K--LPN--- --AGLRSYFD IVEKGQPDEY RTTFYKGSTL AQILEGWRPT LET---LSQR WPSLVEFEND LAKKVGPMSI MKPL--EERI PDI-DSYYEG
LC338003/Dromedary/17C/GpI      ---------- -----MPKNN ETK--LGSYF -K--LPN--- --PGLRSYFD IVRNGQPEIY RTPFAKGKDV QEVLKEWEPY LSR---IADK WPTLMDFEND LRGKVGPMSV MKPL--SERM PDI-DHYYED
LC338004/Dromedary/78C/GpI      KYFMIYKFIE RRLLIMPKNN ETK--FADYF -N--LPN--- --PGLRSYFD IVRKGQPDEY RTPFSKGDSV SKVLKDWQPY VDS---LSDK WPTLVDFEND LKAKVGPMSV MKPL--SERL NDI-DHYYDD
LC338005/Dromedary/101C/GpI     ---------- -----MSKSN ELK--FGEYF -N--LPN--- --PGLRSYFD RTRKGNDEEY RTTFFRGKSL QSILEAWKPT IDS---LESK WPTLLNFEND LAKKVGPLSI QKPL--SERM KDI-ESYYES
LC338006/Dromedary/103C/GpI     ---------- ---------- ELK--FSDYF -N--LPN--- --PGLRSYFD RTRKGNDEEY RTTFYKGRSL SSILKAWKPT LDR---IESK WPTLLDFEND LAKKVGPLSI QKPL--SERM DDI-DSYYES
KU729759/Otarine/PF080915/GpI   ---------- -----MPKNC ETK--FGKSF -I--LPN--- --QGLRSYFD IVRNGQPDVI RAPFAKKENP QKVLKEWMIH LES---LSDR WPTLVDYEKD LAKKVGPMSV MKPL--DDRL TDI-DHYYED
KU729757/Otarine/PF080910/GpI   ---------- ------MSKG ETK--FGSYF -K--LPN--- --PGLRAYFS HVEEGQSDEY RTPFFKGKDL QTVLEEWDHT LSR---IKDE WPTLYEFEND LRKKVGPMSI MQPL--DKRM EDI-DAYYDL
KU729767/Otarine/PF090307/GpI   ---------- -----MPKNC ETK--FGKSF -I--LPN--- --QGLRSYFD NVRNGQPDVI RAPFSKKEDP QKVLKEWMPQ LES---LSDK WPTLVDYEKD LAKKVGPMSV MFPL--EKRM DSI-DNYYDD
MG190029/roe_deer/D38-14/GpI    ---------- -----MPKNN ESKSVIGKYF SS--TAT--- --KGLQSYFG RVVKGQPDIY DTPFAKGEST EELLSRWSAV LES---IDKR WPSLWEFEND MKAKVGPLSV MKPL--RDRM SDV-DHYYDD
AB186898/Human/Hy005102/GpI     ---------- ------MQVA PNV--WSKYF -N--IPN--- --PGLRAYFS NVVSGQPEVY RTPFYKGMSL ESICDEWYKK LVS---IDTQ WPTLMEFEDD LRKKVGPMSV MLPL--KERM SDI-DSYYDS
KY855431/Marmot/HT4/GpI         ---------- ----MRYSDI AVKVDMNDIA -C--LPN--- --PNLKAYLS QNVAGYDVVI RSPFGKGESV DSMLSEWISI LESEL-VEDR YTSLLQFERD KASKVGPMSV RLPL--RERL DDI-VNYYES
KY855428/Marmot/HT1/GpI         ---------- --MKDLKSDA VVKVRMGEFF ----APSAF- --PNAKAHFG RVFRGQPNVY DTPFAKGEST EHMLREWNKT LIS---IKDK WPSLYQFETD MQSKVGPLSV MKPL--KDRI QDI-QQYYEG
KY855430/Marmot/HT3/GpI         ---------- ----MRYSDI AVKVDMDEFA -M--LPN--- --AGLKAYLN RNVTGYDCEV RTPFSKGESV TEVLNEWLNI CEPMLKSEET FASLLDFELA KAKQVGPLSI QRPL--RDRL DDI-VHYYEC
LC110353/Mouse/504/GpI          ---------- ---------- ---------- ---------- ---------- ---------- ---------- ---------- ---------- ---------- MAGKVGPLSV MLPL--SERM QDI-NAYYEG
KR902502/Horse/Equ4/GpI         ---------- ---------- ---------- -----ME--- --RKLSQFLK RQLKPYRHDI TTPL--GDHH PEMLSEVKDM LSA---IEVK YPSLYAYEME MASKVGPLSA RKSL--KDRM PDI-LEYYKG
KJ663816/Human/CDC16/GpI        ---------- -----MPKNC EIK--FEDCF -N--LPN--- --PGLRSYFD IVRRGQPDEY RTTFAKDESV QDVLKKWKPF VDS---LTDK WPTLVDFEND LAKKVGPMSI MKPL--DARM EDI-DAYYDG
KC692366/Fox/Fox_5/GpI          ---------- -KGGYHMPKC ETK--FGNYF -K--LPN--- --PGLRAYFE HVVQGQPDEY RTPFFKGKSL DQVLKDWNRH LIK---INSE WPSLYDFEND LGQKVGPMSI MLPL--EDRM SDI-DSYYDL
KF861773/Porcine/221/04-16/GpI  ---------- -----MPKNN ANK-RSRDYF ASSFASS--- --ANLRAYFG SVVKGQSEVY DTPFARDEST KTLLSKWKAV LKS---IENT WPSLYKYEID LASKVGPMSV RKPL--SERM EDI-EHYYAD
KR902505/Horse/Equ2/GpI         ---------- -----MPKNN EVK--FEDYF -N--LPN--- --PGLKSYFD IVEKGQPDEY RTTFAKGETV QKVLSEWEPI LHS---IHDK WPSLVEFEND LKAKVGPMSI MKPL--KERM EDI-DHYYDD
LC338007/Dromedary/78C/GpII     ---------- ---------- ---------- ---------- ---------- ---------- ----YEDLPI ETILEKVKSI VAKCNKNSPF EKEFNQFEKK NLEKFGPQGK VPPFKSKEFQ EVL-EPIFEP
LC338008/Dromedary/101C/GpII    ---------- ----FTMKSE PINSTLQNYI ----SNN--- --SSLSSYLD SLNRGRQATP RSWLYETRDA KQVLQTWIPI MESANNKTPF GKEFNQFDRK QIEKFGPQGA VPPVLSQECQ EVI-EPLFSP
LC338009/Dromedary/103C/GpII    ---------- ------MKSE PLNSTLQLLV ----RKN--- --NNLSSYLD ALTRGRQATP RSWLYEKEDA NTVLQKWIHI MESANNESPF GELFNQFDLK QVEKFGPQGA IPSVQSKEVK EVI-EPLFSS
KY855429/Marmot/HT2/GpII        MGYQSVKRGT RRKSNTLRKE KTMKEIRIDA QS--LPNLSE IGVGLPLYLS NLSRGRQMTP RSWLFENRDA HVILKQWIRV MKAGNQKSQF ADQLIEFDLK QMEKFGPQGG IPSIDSSECY DVI-EPLYSS
KJ663814/Human/CDC23/GpII       ---------F QRRFKLMKIE SLPKQVSEIV ----LNN--- --SGLKMYLN NLERGRSATP RSWLYEGKSD TEVLQLWLKQ LETVK-ALEY GELVYQFETS QLKKFGSQGE VKPV--KELM DLV-TEGYGQ
AF246940/Human/4-GA-91/GpII     ---------- ------MKSE PVSSTTLQLI ----KCN--- --NSLSSYLQ NLSRGRLATP RSWLYESEKA ESVLQRWIPI MKSANNKSKF GDEFDQFDLK QVEKFGPQGA VPPIDSDACK EVI-EPLFSP
KR902507/Horse/Equ3/GpII        ---------- ------MKIE SLPSEVYDVV ----LNN--- --DGLKMYLN NLERGRSATP RSWLYEGKSD TEVLQAWLTI LETVK-SSEF GELIYQFDTS QLKKWGAQGE VKPI--KELM EIV-TEGFTN
KR902503/Horse/Equ1/GpII        ---------- -------MRQ ALPKELRDVI ----SSN--- --NNLSRYLL SLSKEQAFTD RSWLYEDREP DDVLQAWKEH LAVLENGNTF EREIFQFDTS QEKKWGPQGG HEAF--EEIY QSIVAPQFED
KX884062/peanut_worm/GpIII      ---------- ---------- -MNFQFLDTL SVSKQVE--- --NSVSSNLR RVVSGSEDVL TSPIVKRKAP EDILKGWDEI VKEN--LSAM NADLINLEES NKLKFGPRSI AAPW--GERT DGV-LSYFAE
KX884064/peanut_worm/GpIII      ---------- -----MVKHN QFS--FLDSL DLDKEVK--- --LRLSSNLL NIIQGNDTVL YSPLVKSVKP ESILTGWDKI FKSK--SVKM NKVLHDLEDD NRSKYGPRSI AVPW--VDRR SDV-LSYFES
AP014891/Diatom/GpIII           ---------- ----MVYKTQ NFRYSFISKL NLDSVAV--- --RKLSLLLN SVVSGSNVAQ RSPIGKSMSA ADIVSAWSAI FEKD--SSAL TEGLMRQEMK NKQTIGPRSM AIPW--SLRS SSV-HDYYSS
```

```
                                ....|....| ....|....| ....|....| ....|....| ....|....| ....|....| ....|....| ....|....| ....|....| ....|....| ....|....| ....|....| ....|....|
                                       140        150        160        170        180        190        200        210        220        230        240        250        260
LC338002/Dromedary/15C/GpI      ILLPSE---- -PISDR---- AIKAVISEWS D-V--SGIRL RSDLETVRLM KK----STNS GSPYFTKRRR VSDDTIPVT- ---------L TILGDEVVQC LGPDDRE--- ---------W L--SCAVLGW
LC338003/Dromedary/17C/GpI      ILLPSQ---- -PLDTR---- AIKAVEAEFR K-V--KGLRV RSEAKTVALM KK----STNS GSPYFTKRRN VVDDTVPCVT FVRGNEVDMY LNAFCADRMA NSGVPYEYCI ATKQDQSDNW K--ACAVLGW
LC338004/Dromedary/78C/GpI      ILLSST---- -PVSDK---- AVRAVLSEFN S-I--KGLRV RDQRKTADLM KK----STNS GSPYFSKRKY VLDKTLDVHV YMPNSTRVVQ TLGMRRHQIT PRVSV----- ---------W D--ACAVLGW
LC338005/Dromedary/101C/GpI     ILLDSK---- -PVSNS---- AVAAVIKEWG N-P--RGLLL RSQRKTVDIM KK----STNS GSPYFSKRRA VTGKTLPSR- ---------L DYLGIQTKQF LAETE----- ---------W Y--SAAVLGW
LC338006/Dromedary/103C/GpI     ILLDSK---- -PISNS---- AVAAVIREWG N-P--RGLLL RSQRKTVDIM KK----STNS GSPYFTKRRA VSDKTLPVK- ---------L DYLGIQTKQF LAETE----- ---------W Y--SAAVLGW
KU729759/Otarine/PF080915/GpI   ILLPSK---- -PIDDR---- AVKAAIAEFA G-S--RGLRV RDQKRTIDLM KK----STNS GSPYFTKRRR VVDSTYPC-- ---------Q VYANTMTQVL TSGAE----- ---------Y D--MAAVLGW
KU729757/Otarine/PF080910/GpI   ISLDQT---- -PLDPA---- ALKAAQREWA Q-V--AGLRI RGQNRTVNVM KK----STNS GSPYFTKRKR VVHDTVPTS- --------VY WDGLDAYQDI PSGT------ ---------W N--SCAILGW
KU729767/Otarine/PF090307/GpI   ILLPSV---- -PIDDR---- AVKAVISEFS K-C--RGLRI RNQRKTVDLM KK----STNS GSPYFTRKKR VVDETLPCE- ---------- -VFASDMTQS LDGDI----- ---------Y D--LAAVLGW
MG190029/roe_deer/D38-14/GpI    ILLSSE---- -PIRQS---- AINAVLAEWG S-A--KGLRL RSTERTLNAM KK----STNS GSPYFTKRRR VVEDTVP--- ---------Y TLRWMDRDVY QALNH----- ---------W DGNACAILGW
AB186898/Human/Hy005102/GpI     ISKDQV---- -PFDTK---- AISAAKSEWK G-V--SRLRL RSEVNTVAVM KK----STNS GSPYFSKRKA VVSKTIPCD- ---------V YMDGRYCVMR QNGRE----- ---------W S--GAAVLGW
KY855431/Marmot/HT4/GpI         VVNVDAYE-- -PIDGN---- AIRSYIQEVR RLV--GGMHP KSIDETVWRM KK----STNG GLPTFQKRRE LIPLIK---- ---------- SSRLTRDAQY WDGRW----- ---------Y K--TAAVLGW
KY855428/Marmot/HT1/GpI         ILLPSN---- -PIDPS---- AIKAVISEFN V-V--RGIGI RTTSETIKFM KK----SSNS GTPYFTKRRN VLESGA---- ---------- --NLRILQHS TTTDG----- ---------Y T--NAAILGW
KY855430/Marmot/HT3/GpI         VMDGDEYE-- -PIDGS---- VIRSYIQDVR RAV--GGMHA MGMDEAIRRM KK----STSG GLPEFAKRRT LVPYVA---- ---------- NARLERHEIY WRGHW----- ---------Y K--VSAILGW
LC110353/Mouse/504/GpI          IHLPSA---- -PIDRR---- AIKAVIAEWG R-A--STIRL RSQQQTVDLM RK----STNS GSPFFVKKRT VVDKTVPCR- ---------L HRSSDAVEQF LPG------- ---------W QGEACAVLGW
KR902502/Horse/Equ4/GpI         IDAAQE---- -PIEPE---- AIEATSKFFN I----HNLHL YSPEQAYANM PH----QTNS GYPNWTKRYK AYELEFP--- ---------I SLSDNGTIRS NGEIV----- ---------N D--YPAILGW
KJ663816/Human/CDC16/GpI        VKGHHQ---- -PILDS---- AISAVIQEWS K-S--RGLQV RRQSLTLNLM KK----STNS GSPYFTKRKN VVNKTAFCN- --------VL GSN--SCYVQ YLGAPKFQYL HDPGKFSYTY A--CAAVLGW
KC692366/Fox/Fox_5/GpI          ISKDQV---- -PIPQE---- ALDAALDEWK Q-A--SGLRA RAQKSTVDWM KK----STNS GSPFFTKRRN VVKDTVPCL- --------IT HTYDGNTLQF LGGGTKKFNV ---------Y E--SCAVLGW
KF861773/Porcine/221/04-16/GpI  ILQSAE---- -PLSDR---- AIAATIREFN R-L--RGLRL RSESRTVELM KK----STNS GSPYFTKRRL VTDKTVPSE- --------VY HFRDEVEQHL SSGD------ ---------F T--AAAILGW
KR902505/Horse/Equ2/GpI         ILLSST---- -PVSDK---- AVKAVLSEFK Q-V--KGLTP RSQQKTVDLM KK----STNS GAPYFTKRRS VAKKTLFT-- ---------F CENDDLGTIM WLNSSQDDPR TAGFYASGHF R--ASAVLGW
LC338007/Dromedary/78C/GpII     SKYDNEMALS -QYFKY---- AKPFCEWLFG KRL--QTKRR KSIESVIDDM SDRDTLSSNS GYPRFRRRSV VKDEEI---- ---------- -------QDA LTGKA----- ---------F E--YPAILLG
LC338008/Dromedary/101C/GpII    TEYDDENALK -QYWED---- AHSFAQEAFG KRL--KTKRP RSFNSVIDDM SARDTLVTNS GFPRFRRRKL VKEEEI---- ---------- -------QDA LTGKA----- ---------Y E--YPAIILF
LC338009/Dromedary/103C/GpII    TEYDSEDALS -KYWKD---- AAEFAKVLFG PRL--KTKRP RSAKSVIDDM RARDTLVTNS GFPRFKRRQL VLEQEV---- ---------- -------QDA VAGRA----- ---------Y D--YPAIILF
KY855429/Marmot/HT2/GpII        SEYDDALLLK -KWFSK---- AEEFGRVIFG T-KNCLRRRP LAYTSVVKDM SRRGTLATNS GYPRFTRRRK TAAQEV---- ---------- -------QDA ENLTA----- ---------Y D--YPAIILF
KJ663814/Human/CDC23/GpII       AGTPIPALFR TELWQKAKQN AIKYLIKEIG I-Y--KRLRP RSYDAVVDNM RDRDTLDSNS GFPMFSRRKK PDVLKN---- ---------- -----AYDSI SDGSY----- ---------L E--FPAILLF
AF246940/Human/4-GA-91/GpII     TKYDDEQALQ -HLWHD---- AKRFAEEAFG VRL--LTKRP KSFKRVVDDM RARDTLTTNS GFPRFTRRDS VKGDEI---- ---------- -------QDA SSGLA----- ---------Y E--YPAIILF
KR902507/Horse/Equ3/GpII        AGTPKPAIWN TPMWQQAKTN AIKYLIIDTG L-Y--KRLRP RALSHVVDDM RDRDTLESNS GFPDFGRRKK PEILTA---- ---------- -----AFEAI KSGEF----- ---------W E--FPAIVLF
KR902503/Horse/Equ1/GpII        SPVPKA--FT SELWNQ---- AKAETVAMFK R-AGIRALRP AAYTSVIDNM RARDTLESNS GWPSFARRNV EDVKQQ---- ---------- -----AIQDA ENGK------ ---------W KT-YPAIALF
KX884062/peanut_worm/GpIII      DTLFTP---- -PTVSK---- ---------- --VPRRNLRP ISEAKALGFL KN----STNS GLPYYTKKGK VKEIYS---- ---------- -----TMQSP LLSRK----- ---------- ---DPCVMFT
KX884064/peanut_worm/GpIII      NNMERP---- -PLGLP---- SLR------- -----RNLRP LELDKAIKLL KN----DTNS GLPYYRRKGG LKDIYL---- ---------- -------KEF TNILP----- ---------R E--DPCVMFT
AP014891/Diatom/GpIII           DNNRKL---- ---------- KNNSYHKPYR T----NRLRP IELKKALSFI KN----NTNS GLPSLTRKGM VKDALEK--- ---------- -----SLNHY LSRK------ ---------- ---DPCVLFT
```

```
                                ....|....| ....|....| ....|....| ....|....| ....|....| ....|....| ....|....| ....|....| ....|....| ....|....| ....|....| ....|....| ....|....|
                                       270        280        290        300        310        320        330        340        350        360        370        380        390
LC338002/Dromedary/15C/GpI      RGQEGGPNAN DVKQRVVWMF PYGVNIRELR FYQPAIEAAQ KKG---LV-- --PAWVSMEA VDERITRMFD TKRPEDL-VI CTDFSKFDQH FGQHCQDAAR SIIAALLT-- -KSHHKWLDN TFPVKYSIPL
LC338003/Dromedary/17C/GpI      RGQEGGPSAD DVKQRVVWMF PFGVNINELQ VYQPLIESCQ RFN---LV-- --PAWVSMES VDQRITAMFD TKGKEDL-VV CTDFSKFDQH FNSDMQSAAK DILWRLST-Q DAKWDHWFTN VFPIKYMIPL
LC338004/Dromedary/78C/GpI      RGQEGGPSVE DVKQRVVWMF PFAVNIAELQ VYQPLIESCQ KFN---LV-- --PAWISMES VDRRITDMFD TKGKEDL-VI CTDFSKFDQH FNADMQNAAE AILSGLFT-D NADTRVWLNN IFPIKYAIPL
LC338005/Dromedary/101C/GpI     RGQEGGPTDE DVKQRVVWMF PYAVNICELQ FYQPAIEIAQ KRL---FT-- --PAWVSMEA VDERITKLFD TKGKHDL-VI CTDFSKFDQH FNAHMQEAAK KIISAILG-S DSECRRWLND VFPVKYSIPL
LC338006/Dromedary/103C/GpI     RGQEGGPTDD DVKQRVVWMF PYAVNISELQ LYQPAIEIAQ QRL---FT-- --PAWVSMEA VDDRITKLFD TKGKHDL-VV CTDFSKFDQH FNKDMQDAAK RIISAIMG-S NAECRYWLNE VFPVKYSIPL
KU729759/Otarine/PF080915/GpI   RGQEGGPNPD DVKQRVVWMF PFAANIRELQ VYQPLIESFQ KND---LV-- --PAWVSMDA VDQRITKMFD TKGDDDL-VI CTDFSKFDQH FNPDMQQCAK TILTAMLT-P SQDSRTWLDH VFPVKYNIPL
KU729757/Otarine/PF080910/GpI   RGQEGGPKPE DVKQRVVWMF PFAQNIAELQ VYQPLIEAMQ SRD---LV-- --PAWVGMES VDRAITRMFD TKGPDDL-VV CTDFSKFDQH FNPVCQEVAR EQLGFLFH-G DQGMASWLQN VFPIKYMIPL
KU729767/Otarine/PF090307/GpI   RGQEGGPTSD DVKQRVVWMF PFSVNIRELQ LYQPLIEAFQ RFN---LV-- --PAWVSMEA VDQRITKMFD TKAPDDL-VV CTDFTKFDQH FNPDLQNAAR TILQNLLT-P DRESRTWLDQ VFPVKYAIPL
MG190029/roe_deer/D38-14/GpI    RGQEGGSKED DVKQRVVWMF PFAVNISELR CYQPLIEVAQ KNN---IV-- --SAWVSNDA VDQRRITTLFD TKGKSDL-IV CTDFTKFDQH FNPDMQDAAK RISRIFA-A NSDYANWLEQ VFPIKYMIPL
AB186898/Human/Hy005102/GpI     RGQEGGPKPT DVKQRVVWMF PFAVNIRELQ VYQPLILTFQ RLG---LV-- --PAWVSMEA VDRRITKMFD TKGPRDV-VV CTDFSKFDQH FNPTCQSVAK ELLADLLT-- GQEAVDWLER VFPIKYAIPL
KY855431/Marmot/HT4/GpI         RGQEGGPNPS DVKQRVVFMF PMLVNVHELR VYQTLIEAYQ KAR---LV-- --PAWISNDY VDNIITKLFD TKGDDDL-VI TDFTKFDQH FNAHCQAAAR SILQSLFT-- --GLDEWLDL VYPIKYDIPL
KY855428/Marmot/HT1/GpI         RGQEGGYSKD DVKQRVVWMF PFSVNVQELR VYQPLIEMAQ RHE---IV-- --PAWVSMEC VDHYITKLFD TKAPEDL-VV CTDFTKFDQH FNQHLVETAA DVLRSVFN-- -STINGWLED VFPIKYNIPM
KY855430/Marmot/HT3/GpI         RGQEGGPKPG DVKQRVVFMF PMLVNLYESQ VYNVLIEAYQ KAG---LV-- --PAWISNDA VDLTITKLFD SKEESDP-VI VTDFTKFDQH FNVQCQNAVR SVLQSTFT-- --EINDWLDA VYPIKFEIPL
LC110353/Mouse/504/GpI          RGQEGGHSKE DVKQRVVWMF PFAVNVAELQ FYQPAIEYAQ RFG---LV-- --PAWVSNDE VDKVMTRLFD TKADTDL-VV ATDFTKFDQH FNSSLQDAAQ TIIEARLTHD GASAAGWIRD IFPIKYMIPM
KR902502/Horse/Equ4/GpI         RGQEGGPSLD DTKQRCLFMF PFTYNVLEAQ FYQPFTIAAQ VNG---TV-- --PPWNGRHE VDVGVTRLF- -RSTKDP-IM CTDFSGYDQH FGKSVQDATR TLIASTLR-D DEYSRLWLNY IFPIKYNIPL
KJ663816/Human/CDC16/GpI        RGQEGGPAPD DVKQRVVWMF PFAVNLCELQ VYQPLIESAQ RFN---TV-- --PAWVSMEM VDQEITHLFD TKGKDDL-VV CTDFTKFDQH FGIPMQEAAK KILSAIFT-D NEASRWWMNW IFPIKYDIPL
KC692366/Fox/Fox_5/GpI          RGQEGGPNPE DVKQRVVWMF PFAVNIRELQ VYQPLIIAAQ NHN---IV-- --PAWVGMDA VDQRITQMFD TKDPDDY-VI CTDFSKFDQH FNPSCQDAAK YMLEHLFK-G DKSMKDWLQD VFPVKYDIPL
KF861773/Porcine/221/04-16/GpI  RGQEGGPTAA DVKQRVVWMF PYAVNIAELQ LYQPFIEAAQ SSI---SS-- --QLGLALTR LINGSLPCST QRQTNDL-VV CTDFSKFDQH FNAEMQAAAL RILRACME-P SMTSTNWLND VYPIKYMIPL
KR902505/Horse/Equ2/GpI         RGQEGGPEPG DVKQRVVWMF PFSVNINELQ VYQPLIESCQ KFD---LI-- --PAWVSMES VDRRITKMFD SKGKDDV-VI CTDFSKFDQH FNSDMQDAAY SIISQILN-G GTAAQWWLKE IFPIKYNIPL
LC338007/Dromedary/78C/GpII     RHYHG----- --KLRPVWMY PMSMNLLEYQ FSQVIQDSLR NSPN-PQVRA HLSPWEGYED VKRTLTSYWH GQT-----IV GGDTTKMDAH MRRAQNRLCY ELVKGLFQ-- -KHEWDDLYK VIMRVNDIPI
LC338008/Dromedary/101C/GpII    RQYNG----- --KLRPVWMF PMSANLVEFT FTQEIQDSLQ NSPA-AWIQK YLSPWKGYED VKLTLTEQWK GQ------VD GGDTTKMDAH MRPAQIRLVY EIVKWLFQ-- -EKYWDELKK SLNHICNIPL
LC338009/Dromedary/103C/GpII    RHYNG----- --KLRPVWMF PMSQNLIEFQ YSQVIQDSLQ QSPT-GWVRE YLSPWKGYED VKQTLTAYQP KEIN-----IV GGDTTKMDAH MRPAQIRLVF EIVNWLFQ-- -ESYWDALHR CLIHICEIPL
KY855429/Marmot/HT2/GpII        RFYHG----- --KLRPVWMF PMSVNLIEAS FAVQIQDCLA QSPV-KWVRD YCTPWKGFDH VKSVLTEQWP QKAA----IV GGDTTKMDAH MRPAQIKLVF EIVKWLFQ-- -RKYWNNLEK SLMYICSIDL
KJ663814/Human/CDC23/GpII       RNYNQ----- --KTRVVWMY PMSTNMVEGS FTQPLKEAIT NSD---LQ-- FFAPWRGYEH VLSRITKHYN DGEF----IS ASDFSHADAH FTKWAMLEVY DVIKYAFQ-- -EQYWPSLKQ SMLHVNSIPL
AF246940/Human/4-GA-91/GpII     RQYNG----- --KLRPVWMF PMSANLIEFS FAQVIQEELQ SSKTCKWIRD YLSPWRGFEY VKETLTATWP HPAE----IV GGDTTKMDAH MRPAQIRLVF EIVKWLFQ-- -REYWPKLYQ SLIHICEIPL
KR902507/Horse/Equ3/GpII        RNYNQ----- --KTRIVWMY PMATNIYEGS FTQPLKEALM KSD---LD-- FIAPWRGYDH VLARLSKLYG TGEF----LS ASDFSHTDAH FTKWAILEVY DVIKYAFQ-- -EQYWDELKR SMLHVVEIPL
KR902503/Horse/Equ1/GpII        RRYNN----- --KTRLVWMY PMSVNLVEAS FFQPLQTAIL KTH---LAKE FFAPWVGFEQ VRVRVTEEYQ SGRF----VS ASDFSATDQH FRLSTSLEVY DVLAELFM-- -PQYREPLKE SIQCMHNIPL
KX884062/peanut_worm/GpIII      RTQEQR---- --KTRTVWGY PMADTLREMQ YYEPILGYQR KLN---WR-- --NSLSGPDA VNRKMSDLVT KAISSDQYLL SIDFSAYDAS VKTTLQKASF DYFKSLYQ-- -ADYHEEIDD IATRFNTIGL
KX884064/peanut_worm/GpIII      RTQEGK---- --KTRTVWGY PMADTVNEMC VYSPLLGYQR KLN---WR-- --SCLNSPDA VNIEITKLID KALKQDVSLL SIDFSAYDAS LDRPLQKAAF EYISKLFQ-- -PSYEELINN LFNRFNTIGL
AP014891/Diatom/GpIII           RTQE------ DFKTRNVWGY PAADVVNEMR YYRPILDYQK GLP---WR-- --TALVSPEA TDLAITHIMK EARENGFFLV SIDFSSYDAT VSSEKQTRAF TYVKDLFQ-- -SAYSEEIDY IAERFNTIGL
```

```
                                ....|....| ....|....| ....|....| ....|....| ....|....| ....|....| ....|....| ....|....| ....|....| ....|....| ....|....| ....|....| ....|....|
                                       400        410        420        430        440        450        460        470        480        490        500        510        520
LC338002/Dromedary/15C/GpI      AYDFGK---- --IRRGKHGM GSGSGGTNFD ETLTHRALQY EAAQSVGSFL NPNS----QC LGDDGVL--- TYPGITVEDV VRSYTAHGLE MNPDKQYVST TDCTYLRRWH HTDYR-IN-- NVCV------
LC338003/Dromedary/17C/GpI      MYDLGE---- --LRTGSHGM GSGSGGTNAD ETLAHRALQY EAALANSSKL NPNS----QC LGDDGVL--- TYPGITVEDV VRSYTAHGQE MNESKQYASK HDCTYLRRWH HEDYR-VD-- GICV------
LC338004/Dromedary/78C/GpI      AYDYGK---- --IRYGKHGM GSGSGGTNAD ETLAHRALQY EAALANNARL NPNS----QC LGDDGVL--- TYPGITVEDV VQSYSAHGQE MNESKQYAST HDCVYLRRWH HEDYR-EG-- GVCV------
LC338005/Dromedary/101C/GpI     AIDMDS---- --ILTGYHGM GSGSGGTNFD ETLTHRSLQY EAAQSQRKML NPNS----QC LGDDGIL--- SYPGITVEDV THSYSSHGQD MNVDKQYASE HDCTYLRRWH HDAYR-VD-- GVCV------
LC338006/Dromedary/103C/GpI     AIDMNR---- --IITGYHGM GSGSGGTNFD ETLTHRSLQY EAAQSAKQML NPNS----QC LGDDGIL--- SYPGINVEDV TRSYSAHGQD MNVEKQSASE HDCTYLRRWH HDEYR-IN-- GVCV------
KU729759/Otarine/PF080915/GpI   TYDYGK---- --IRCGSHGM GSGSGGTNAD ETLVHRTLQY EAAQSVNQKL NPFS----QC LGDDGVL--- TYPGIQVEDV VRAYSSHGQE MNTSKQYASK HDCTYLRRWH HTGYR-VD-- GVCV------
KU729757/Otarine/PF080910/GpI   AYDMDK---- --IRLGRHGM ASGSGGTNAD ETLLHRTLQH EAAILNHSTL NLNS----QC LGDDGVL--- TYPGIKVEDV VRVYCSHGQE MNVDKQYAST HDCTYLRRWH HKDYR-VN-- GVCV------
KU729767/Otarine/PF090307/GpI   AYDYNK---- --VRCGKHGM GSGSGGTNAD ETLVHRSLQY EAAMQANRRL NPYS----QC LGDDGVL--- TYPGITVDDV VRTYASHGQV MNTSKQYASK HDCTYLRRWH HDQYR-VD-- GVCV------
MG190029/roe_deer/D38-14/GpI    MYNYGL---- --IRYGKHGM GSGSGGTNAD ETLSHRALQF EAAQRAGKLL NPNS----QC LGDDGIL--- TYPGITVEDV IGTYQSHGLE MNASKQYVST NDCTYLRRWH CAQYR-VN-- GVCV------
AB186898/Human/Hy005102/GpI     AYNWGE---- --IRYGIHGM GSGSGGTNAD ETLVHRVLQH EAAISHHTTL NPNS----QC LGDDGVL--- TYPGISAEDV MQSYSRHGLD MNLEKQYVSK QDCTYLRRWH HTDYR-VD-- GMCV------
KY855431/Marmot/HT4/GpI         LNRWGE---- --ITIGSHGM GSGSGGTNAD ETLVHSVLQR ECAKMAGSQL NPYS----MC LGDDGII--- SFPGCSVESV TNSYTRHGLD MNLEKQYYST DDCVYLQRWH SKHYR-VE-- GICA------
KY855428/Marmot/HT1/GpI         LISNSQ---- --LVKGRHGM GSGSGGTNAD ETLAHRALQY EAARKIGSRL NPNS----QC LGDDGIL--- SYPGITVEDV VRCYSAHGLE MNTSKQYAST QDCVYLRRWH HEKYR-IN-- HVCA------
KY855430/Marmot/HT3/GpI         MTRWGE---- --VTMGAHGM GSGSGGTNAD ETILHTLLQR ESAKMAGKQL NPFS----MC LGDDGIL--- SYPGCSVEHV THCYTRHGLD MNETKQSSST DSCVYLQRWH SQHYR-VN-- GRCV------
LC110353/Mouse/504/GpI          AYNFGE---- --IRRGEHGM GSGSGGTNFD ETIAHRALQY EAAQRNRVKL NPNS----QC LGDDGIL--- SYPGITVEDV VDTYTSHGLE MNDQKQYAST HDTVYLRRWH HKDYR-VD-- GRCV------
KR902502/Horse/Equ4/GpI         MISTRR---- --TLTGSHGL GSGSSGTSGD GTIGHKILQF EAALRNGANL NPNS----MV LGDDGII--- TFKGISAKKV MYSYTNHGMR MNSDKQSISD EYVIFLRRWH SKRYRSAS-- GLNV------
KJ663816/Human/CDC16/GpI        AYDYGK---- --VRCGKHGM GSGSGGTNAD ETLSHRALQY EAAIKAKKKL NPHS----MC LGDDGIL--- SYPGITVDDV TRSYAVHGLE MNPDKQSASA HECTYLRRWH HEDYR-VD-- GVCV------
KC692366/Fox/Fox_5/GpI          AYDFGK---- --VRTGKHGM ASGSGGTNAD ETLVHRALQY EAAIRAGTKL NPHS----QC LGDDGVL--- TYPGCDVDDV VSTYSSHGLD MNPDKQYVSK QDCVYLRRWH HTRYR-RD-- GVCV------
KF861773/Porcine/221/04-16/GpI  AYDEGK---- --VRFGKHGM GSGSGGTNCD ETLAHRALQY EAAQSSGVKL NPNS----QC LGDDGVL--- TYPGITVEDV VKAYKSHGLE MNLDKQYAST QDCTYLRRWH HKDYR-QD-- GICV------
KR902505/Horse/Equ2/GpI         AYNYGM---- --IRFGKHGM GSGSGGTNAD ETLAHRALQY EAAINSGTLL NPYS----QC LGDDGVL--- TFPGITVDKV VDTYTAHGQE MNIDKQYVSK HDCTYLRRWH HDQYR-EN-- GICV------
KY855429/Marmot/HT2/GpII        LWKFDKETNQ YVKLEGVHGL ASGSGSSWTQLA ETVLQLFMAY IQGTQG---- --------QG IGDDFYW--- -TTDMQADAL VEYLGEFGLP ANPAKQSVSE DDLIFLQRYF HQGFFSRESG TVLG------
KJ663814/Human/CDC23/GpII       IIGSDT---- --WIIGDHGV ASGSNWTNDV ETYMDFIAEQ YLTLL-GLVK EPDT-----A IGDDISHRRD TYLETLAELL ADEYKKMNFD VNAQKVTNER DWVKYLQRLT VRGYYSRR-- TVVVKGKEYP
LC338007/Dromedary/78C/GpII     LVGPND---- --MIQGYHGL ASGSGWTQLL ETLLVLFIAW LHKIKHS--- --------MG IGDDFTF--- -LANITAEQL VKWLAEFGLP ANALKQTVSK LVLDFLQRCN AQGFFSREDP NVLG------
LC338008/Dromedary/101C/GpII    LYSKTD---- --AYVGVHGL ASGSGWTQLT ETVLQMFMAW KRGVIG---- --------QG IGDDFYW--- -MADMSAEEL VDYLGEFGLP ANPAKQTVDT VTLTFLQRMN HQGFFSRENQ ACLG------
LC338009/Dromedary/103C/GpII    LYSQTD---- --TYVGTH-- ---------- ---------- ---------- ---------- ---------- ---------- ---------- ---------- ---------- ---------- ----------
AF246940/Human/4-GA-91/GpII     LYSTEK---- --QYCGVHGL ASGSGWTQLT ETVLQLFMAW RRGVTG---- --------QG IGDDFYW--- -LADMDAKEV VDYLGQYGLP ANPTKQSVGT ETLTFLQRYF RQGFVSREAG GVFG------
KR902507/Horse/Equ3/GpII        IIGENS---- --WIIGDHGV SSGSNWTNDV ETYMDFIIAQ YLTLL-KLVK EPCM-----A IGDDISHRRD SYLPDLDEQI AQVCQSMGFD VNAEKVTNQQ DWVKFLQRLT IRGFNSER-- TYSVEGVEYP
KR902503/Horse/Equ1/GpII        VISPTE---- --YIVGEHGV ASGSNWTNFI ETVFDFILSN YVELLSSNSQ EVHYWSGLYG IGDDMSWFCN KYDPKFSEWL SDVGQSVGQE INAEKVTNDP DKVKTLQRLF QRGYRRPD-- GLLR------
KX884062/peanut_worm/GpIII      VTPDG----- --IIDGPHGV PSGSTFTNEV DSVAQYLIAT EYGLS----- SDNF----DI QGDDGA---- -YVVSDPDSL KDYFRKYNLV VNDDKSYISK DYLIYLQNLY HKDYM-RD-- GIIG------
KX884064/peanut_worm/GpIII      VTPDG----- --ILSGHHGV PSGSTFTNEV DSIIQYLIAL DYGLEDSSMQ ---------I QGDDGV---- -YCVQDGTAL AEHFKSFGLN VNFEKSYYSK DYCVYLQNLY HNDYR-DSSG KIC-------
AP014891/Diatom/GpIII           VTPDG----- --ILTGKHGV PSGSTFTNEI DSIVQYLIAK DFGIS----- DDHM----NI QGDDGV---- -YCVKDPSAL LKHFEENGLK VNYEKSLISD CECLFLQNYY SFDYI-RN-- GQIK------
```

```
                                 ....|....| ....|....| ....|....| ....|....| ....|....| ....|....| ....|....| ....|....| ....|....| ....|....| ....|....| ....|....| ....|....|
                                        530        540        550        560        570        580        590        600        610        620        630        640        650
LC338002/Dromedary/15C/GpI       ---GVYSTYR ALGRLAEQER FYD---PEEW GPKMVALRQL SIIENVKWHP LREAFADYCM KGDKFRLGI- -DIPGFLDNI EQEAREAIEH MPDFLGYTKS MQKVN---AN -GAGISKWWI VQYLKSKV--
LC338003/Dromedary/17C/GpI       ---GVYSTYR ALGRLMEQER YYD---PDVW SNKMVALRQL SIIENVKWHP LRDQFAEFCM ERDKYRLGI- -DIPGFLDDI DSISREAIDL MPDFLGYTKS MQP------- -EVGLSQWWI VQYLKSKR--
LC338004/Dromedary/78C/GpI       ---GVYSTYR ALGRLMEQER YYD---PDKW SNKMVALRQL SIIENVKYHP LRDQFADFCM KRDKYRLGI- -DIPGFLDDI DNIAKESIDL MPDFLGYTKS MNKDS----- -ETGLSTWWI VKYLKSKR--
LC338005/Dromedary/101C/GpI      ---GVYSTYR ALGRLCEQER YYD---PEIW GPKMVALRQL SIIENCKYHP LREEFVNFCM KGDKFRLGI- -DIPGFLDDI DTIASEATDL MPDFLGYTKS LQQGT----- -DTGISSWWI IQYLKSLR--
LC338006/Dromedary/103C/GpI      ---GVYSTYR ALGRLCEQER YYD---PEIW GPKMVALRQL SIIENCKYHP LREEFVNFCM KGDKFRLGL- -DIPGFLDNI DSLAKEATDV MPDFLGYTKS LQPKS----- -ATGLSSWWV VNYLKSIR--
KU729759/Otarine/PF080915/GpI    ---GVYSTCR ALGRLMYQER FYD---PEVW GPKMVALRQL SILENVKWHP LRDQFAEFCM KRDKYRLGI- -DIPGFLVNI EQEAKEAIDL MPDFLGYTKS MQMGD---SP RQTGIEDWWI VNYLKSKQ--
KU729757/Otarine/PF080910/GpI    ---GVYSTMR ALGRLAEQER YYD---PEIW GPKMVALRQL SILENVKWHP LREEFVDFCM KGDKFRLGL- -DIPGFFDHI EREAKDAIDY MPDFLGYTKS LQNDGNP--- -SCGIKDWWI VNYLKSKR--
KU729767/Otarine/PF090307/GpI    ---GVYSTCR ALGRLMYQER FYD---PEVW GPKMVALRQL SILENVKYHP LRDQFADFCM ARDKYRLGI- -DIPGFLDNI ESIAKESIEN MPDFLGYTKS LQNET---DP -SAGISNWWI VNYLKSK---
MG190029/roe_deer/D38-14/GpI     ---GVYSTFR ALGRLRYLER YMD---PEIW NPKTVALRQL SILENVKYHP LRDQFVEFCM KRDKYRLGI- -DIPHFLDDL PQIIKEVNDY MPDFLGYTKT LQSEG---DP -TYGIADWWI VKYLRSRQNV
AB186898/Human/Hy005102/GpI      ---GVYSTMR ALGRLAMQER YYD---PDVW GEKMVTLRYL SIIENVKWHP LKEEFLDFCI KGDKTRLGL- -GIPGFLDNI AGEAQKAIDM MPDFLGYTKS LQYDGDLRRN AAAGIENWWV VQALKSRR--
KY855431/Marmot/HT4/GpI          ---GVYATCR ALGRLCYQER YYD---PEYW GREAVAMRQL SIIENIKWHP LREKFADYCM KRDIYRLGL- -DIPGFIDQI DATWWNLNDH IQDFLSYSTM AGMS------ -ITPPSKWWI TSYLKSKA--
KY855428/Marmot/HT1/GpI          ---GVYSTMR ALGRLRYLER YMD---PRVW GPKAVALRQL SILENVKYHP LREQFAEFCM KRDKYRLGI- -DIPGFLTNI EDEVKRVVDY MPDFMGYVKT LQNSE----T DGFGIRKWWI YQYLMNYNA-
KY855430/Marmot/HT3/GpI          ---GVYSTCR ALGRLCEQER YYD---PQLW SREMVAIRQL SILENIKWHP LREQFADFCI TRDIYRLGI- -DIPGFLDNL SALVSKANDQ VQDFISYSSS VGEG------ -VTNPDGWWI VSYLKSKA--
LC110353/Mouse/504/GpI           ---GVYSTMR ALGRMRYLER YMD---PEIW DAEAVELRWY SILNNLEYHP LREQFVDFLH ---------- ---------- ---------- ---------- ---------- -GKG------ ----------
KR902502/Horse/Equ4/GpI          ---GVYPVTR AIGRLMHVER PTD-----KW TREDHIMRTL SICENFKYHP LRSEVLRYII QKDKFRLGL- -DIPGFIEGL KYKSTSDV-- -LQYLNYNQQ LEYAS---S- -HTGITSWWC IKELISML--
KJ663816/Human/CDC16/GpI         ---GVYSTYR ALGRLMEQER YYD---PDNW GPKMVALRQL SIIENVKWHP LRDKFAHFCM LRDKYRLGI- -DIPGFLDNI EVEAKKAIEV MPDFLGYTKS MQTSD---GE -YSGISQWWI VQFLKKYKEG
KC692366/Fox/Fox_5/GpI           ---GVYSTNR ALGRLCEQER YYD---PEVW GPQMVALRQL SILENVKYHP LNEEFVDYCM KGDKYRLGL- -DLPGFFDNL ERYAKQATDY MPDFLGYTKS LQNEG--RDG VRTSISDWWI VNYLKSKR--
KF861773/Porcine/221/04-16/GpI   ---GVYSTCR ALGRLRYLER YQN---PKYW DAKAVALRQL SILENVKYHP LKEQFVDFCM KRDKYRLGL- -DIPHFFDDL RAITEQKIDD MPDFLGYTRT LQSGG---DP -AGGIENWWI VKYLKSK---
KR902505/Horse/Equ2/GpI          ---GVYSTYR ALGRLMEQER FYD---PDVW SAKMVALRQL SIIENVKYHP LRAEFADFCM KRDKYRLGI- -DIPGFLDNI EGIAQEAIDL MPDFLGYTKS MTKD------ -QTGLSQWWI VNYLKSKR--
LC338007/Dromedary/78C/GpII      ---AYYSIIR MANSDFLPEK FHN---PKQW NSDMFCVRHY MITENGVDDP GWEEYVLWTA KGQKDLINFA KKSASELNRI QEEARLIPGL NPSYNQEKR- ---------- -LKPLSEFAS IKLVRDSL--
LC338008/Dromedary/101C/GpII     ---AYYPTIR ALNSMLNPEK FHK---PKDW SSDMFCIRNY MILENCVDNP CFDEFVKFVC RGQKDMIPFA KRENASLNAV QAQARLLP-- ---------- ---------- ---------- ----------
LC338009/Dromedary/103C/GpII     ---------- ---------- ---------- ---------- ---------- ---------- ---------- ---------- ---------- ---------- ---------- ---------- ----------
KY855429/Marmot/HT2/GpII         ---AYYPTIR ALGSMIYPER FHD---PKIW NSDMFCIRNY MILENCVDDP CFDEFVKFVV RGQRDMSSFA KKSARDLDTL DRIARQVPGL SPTYNQEKR- ---------- -DKPLSCFAS IRVAKML---
KJ663814/Human/CDC23/GpII        LLRGIYSTIR ALNSSLNPEK FHS---PKIW SKDMFAVRQF TILENCIDHP LFEELVKFVC DGHPYLIPFA KQTNEQINRA QQESRHIPGL NPTYNQEKR- ---------- -DKPLATFAS INLARKL---
AF246940/Human/4-GA-91/GpII      ---AYYPTIR ALNSLLQPEK FHK---PKDW SSDMFCIRNY MILENCVDDP CFEEFCKFVA HGHKDMITFA KKSDTELSRI QKKSRLVPGL NPSFNQEKR- ---------- -EKPLSSFAS IRFVKEL---
KR902507/Horse/Equ3/GpII         LLRGIYSTIR ALNSSLNPEK FHS---PKLW SKDMFAVRQF MILENCIDHP LFVQFVKFVC AGNPYLVKFA KLKNEQIDKK WAQSRILPGL NPTYNQEKR- ---------- -DKPLSTYAA IAIARSL---
KR902503/Horse/Equ1/GpII         ---GVYSTIR ALKSSVYPEK FHN---PKLW SSDMFCARQF MILENCVDHP LFEDFVKFVC AGQKDLIPFA HKTRAQLDEI NRKTKLLPGL NTTYNQERR- ---------- -ESSLADFAS IRIARNL---
KX884062/peanut_worm/GpIII       ---GIYPTYR ALNRIVYLER FTDFLEDDLK GQDYFSLRTI SILENCKFHP LFEELVKYVA SLDKYKLKY- -SNSGLAKYT QRASQSSG-- VAGIFKYRYE DD-------- -PKGLNNFDT VKLLKEL---
KX884064/peanut_worm/GpIII       ---GIYPTYR ALNRLIHPER FVDFEDIKIS GRDYFSIRSI CILENCKYHP LFEDLVKYIY KLDKYNLKF- -SRDSLFRFV QNRKQESG-- VQGIFNYRYE DD-------- -VSGIDSFET MKVLNKL---
AP014891/Diatom/GpIII            ---GIYPTYR ALCRILYPER YTNYSVEGIA GASYNSIRTI SILENCSQHP LFREFVTFIY SLDKYCLSF- -ESVELSKYI KQNVDSTG-- TEGLFNYRRG LD-------- -LKGIYKFKT VQLINELNSG

                                 ....|..

LC338002/Dromedary/15C/GpI       -------
LC338003/Dromedary/17C/GpI       -------
LC338004/Dromedary/78C/GpI       -------
LC338005/Dromedary/101C/GpI      -------
LC338006/Dromedary/103C/GpI      -------
KU729759/Otarine/PF080915/GpI    -------
KU729757/Otarine/PF080910/GpI    -------
KU729767/Otarine/PF090307/GpI    -------
MG190029/roe_deer/D38-14/GpI     KKSVA--
AB186898/Human/Hy005102/GpI      -------
KY855431/Marmot/HT4/GpI          -------
KY855428/Marmot/HT1/GpI          -------
KY855430/Marmot/HT3/GpI          -------
LC110353/Mouse/504/GpI           -------
KR902502/Horse/Equ4/GpI          -------
KJ663816/Human/CDC16/GpI         VTYMRSE
KC692366/Fox/Fox_5/GpI           -------
KF861773/Porcine/221/04-16/GpI   -------
KR902505/Horse/Equ2/GpI          -------
LC338007/Dromedary/78C/GpII      -------
LC338008/Dromedary/101C/GpII     -------
LC338009/Dromedary/103C/GpII     -------
KY855429/Marmot/HT2/GpII         -------
KJ663814/Human/CDC23/GpII        -------
AF246940/Human/4-GA-91/GpII      -------
KR902507/Horse/Equ3/GpII         -------
KR902503/Horse/Equ1/GpII         -------
KX884062/peanut_worm/GpIII       -------
KX884064/peanut_worm/GpIII       -------
AP014891/Diatom/GpIII            -------
```
